# Environmental factors influencing potential distribution of *Schisandra sphenanthera* and its accumulation of medicinal components

**DOI:** 10.3389/fpls.2023.1302417

**Published:** 2023-12-12

**Authors:** Jingjing Shang, Qian Zhao, Pengdong Yan, Mengdi Sun, Haoxuan Sun, Huizhen Liang, Dezhu Zhang, Zengqiang Qian, Langjun Cui

**Affiliations:** ^1^ National Engineering Laboratory for Resource Development of Endangered Crude Drugs in Northwest China, The Key Laboratory of Medicinal Resources and Natural Pharmaceutical Chemistry, The Ministry of Education, College of Life Sciences, Shaanxi Normal University, Xi’an, China; ^2^ Henan Sesame Research Center, Henan Academy of Agricultural Sciences, Zhengzhou, Henan, China; ^3^ Shaanxi Panlong Pharmaceutical Group Limited by Share Ltd, Shangluo, Shaanxi, China

**Keywords:** medicinal plant, climate change, maximum entropy modeling, suitable habitats, secondary metabolites

## Abstract

Schisandrae Sphenantherae Fructus (SSF), the dry ripe fruit of *Schisandra sphenanthera* Rehd. et Wils., is a traditional Chinese medicine with wide application potential. The quality of SSF indicated by the composition and contents of secondary metabolites is closely related to environmental factors, such as regional climate and soil conditions. The aims of this study were to predict the distribution patterns of potentially suitable areas for *S. sphenanthera* in China and pinpoint the major environmental factors influencing its accumulation of medicinal components. An optimized maximum entropy model was developed and applied under current and future climate scenarios (SSP1-RCP2.6, SSP3-RCP7, and SSP5-RCP8.5). Results show that the total suitable areas for *S. sphenanthera* (179.58×10^4^ km^2^) cover 18.71% of China’s territory under the current climatic conditions (1981–2010). Poorly, moderately, and highly suitable areas are 119.00×10^4^ km^2^, 49.61×10^4^ km^2^, and 10.98×10^4^ km^2^, respectively. The potentially suitable areas for *S. sphenanthera* are predicted to shrink and shift westward under the future climatic conditions (2041–2070 and 2071–2100). The areas of low climate impact are located in southern Shaanxi, northwestern Guizhou, southeastern Chongqing, and western Hubei Provinces (or Municipality), which exhibit stable and high suitability under different climate scenarios. The contents of volatile oils, lignans, and polysaccharides in SSF are correlated with various environmental factors. The accumulation of major secondary metabolites is primarily influenced by temperature variation, seasonal precipitation, and annual precipitation. This study depicts the potential distribution of *S. sphenanthera* in China and its spatial change in the future. Our findings decipher the influence of habitat environment on the geographical distribution and medicinal quality of *S. sphenanthera*, which could have great implications for natural resource conservation and artificial cultivation.

## 1 Introduction

Medicinal plants are a bioresource that provides pharmaceutically active components. The distribution and growth of medicinal plants are dependent on a range of environmental factors, such as geographical location, climate, and soil conditions (Shan et al., 2022; Feng et al., 2023). Various medicinal plant species show distinct habitat preferences. For example, *Scutellaria baicalensis* Georgi is tolerant to low temperature and drought, with hindered growth in hot and humid regions (Ji et al., 2021). *Atractylodes lancea* (Thunb.) DC. is also widely distributed in arid regions (Han et al., 2020), in contrast to *Paris yunnanensis* Franch. that generally favors humid climate zones with concentrated precipitation (Luo et al., 2017). Even cultivars of the same medicinal plant species (e.g., *Anemarrhena asphodeloides* Bunge.) vary in yield and quality across regions (Li et al., 2015). Therefore, the formulation of effective strategies for the exploitation and utilization of medicinal plants necessitates accurate prediction of their potentially suitable habitats.

Ecological niche modeling (ENM) has been widely used to predict the potential distribution of various species and assess their habitat suitability in the natural environment (Blank and Blaustein, 2012). Owing to the development of geographic information systems (GIS), the potentially suitable habitats for a given species can be predicted by analyzing its regional environmental conditions based on the correlation between environmental factors and species occurrence data (Title and Bemmels, 2018). Among the ENM methods, maximum entropy (MaxEnt) modeling has an outstanding predictive power. In particular, the MaxEnt method can predict the distribution of potentially suitable habitats for a specific species in the past and future based on climate data. This would help identify the areas of low climate impact (i.e., low impact areas) for the target species (Xu et al., 2019). MaxEnt model can handle large-capacity data, and can still show excellent prediction ability in the case of small sample size and spatial deviation of distribution point data (Graham et al., 2008). As one of the most popular modeling approaches, the MaxEnt method has been successfully used in the prediction of potentially suitable areas for various species and the conservation planning of medicinal plants (Kaky et al., 2020; Chen et al., 2021).

Medicinal plants synthesize a variety of secondary metabolites (SMs) in response to their habitat environments. Natural SMs with diverse functions enable the plants to defend against pathogenic attacks and environmental stresses (Zhou et al., 2010). Given their remarkable biological activities, many SMs are used as active components in pharmaceutics, cosmetics, fine chemicals, nutraceuticals, and food supplements (Yang et al., 2013). The composition and contents of SMs are quality indicators that determine the therapeutic effects of medicinal plants (Yuan et al., 2020). Farmers pursue high yield and quality (measured by the levels of major SMs) when growing medicinal plants. Importantly, the accumulation profile of SMs in plants varies with specific local environment, in addition to plant genotype. For example, temperature and sunshine duration respectively affect the accumulation of polysaccharides and total alkaloids in the traditional Chinese orchid herb (*Dendrobium officinale* Kimura & Migo) (Yuan et al., 2020). Volatile organic compounds accumulate in lavender (*Lavandula angustifolia* Mill.) mainly depending on the latitudinal gradient (Demasi et al., 2018). In contrast, the SM profile of the bulk phloem of lodgepole pine (*Pinus contorta* Douglas ex Loudon) shifts with elevation (Mullin et al., 2021).

*Schisandra sphenanthera* Rehd. et Wils. is a perennial medicinal plant that is primarily found in western and southern China. It prefers shade and grows well under forest canopies. *S. sphenanthera* develops a solid lateral root and plays a positive role in soil and water conservation (Huang et al., 2021). Its dry ripe fruit, known as Schisandrae Sphenantherae Fructus (SSF), is one of the most famous traditional Chinese medicines and has been used to treat hepatitis, Alzheimer’s disease, osteoporosis, and insomnia (Li et al., 2022). SSF also has wide application in food and cosmetics (Tao et al., 2020). Lignans are the major bioactive components in SSF, and the content of schisantherin A is considered to be a standard indicator for quality evaluation (The State Pharmacopoeia Commission of China, 2020). Lignan components, such as schisantherin and schisanhenol, show pharmacological effects exemplified by hepatoprotective and antiviral activities (Yang et al., 2010; Sowndhararajan et al., 2018). Water-soluble polysaccharides from SSF also exhibit beneficial effects, including antioxidant, immunomodulatory, hepatoprotective, antitumor, and hypolipidemic activities (Zhao et al., 2013). Additionally, volatile oils are the major pharmacological components of SSF that could protect islet B cells and lower blood sugar (Song et al., 2007).

Nowadays, the herbal medicine SSF is mainly harvested from the wild resources of *S. sphenanthera*. In recent decades, it has been challenging to meet the growing market demand for SSF with wild resources only. The development of sustainable conservation and management strategies for *S. sphenanthera* calls for a holistic understanding of its potential geographical distribution and habitat preferences (Azareh et al., 2019). Moreover, climate change reportedly has significant implications for the habitat requirements of various species (Peng and Guo, 2017; Li et al., 2020a). Hence, ascertaining whether climate change affects the habitat suitability for *S. sphenanthera* is another critical issue related to its economic value and ecological significance (Zhang et al., 2020). So far, the ecological distribution of *S. sphenanthera* has been insufficiently investigated, and it is necessary to identify the priority areas where this medicinal plant should be cultivated under climate change.

In the present study, an optimized MaxEnt model was adopted to predict the distribution patterns of potentially suitable areas for *S. sphenanthera* under current and future climatic conditions. The purposes of the present study were to: (1) predict the potential geographical distribution of *S. sphenanthera* and identify its low impact areas under different climate scenarios and (2) determine the major environmental factors influencing the accumulation of medicinal components in SSF across regions. Results of this study could provide guidance for the exploitation and conservation of wild *S. sphenanthera* resources, as well as for the promotion of artificial cultivation over large scales.

## 2 Materials and methods

### 2.1 Prediction of potentially suitable habitats

#### 2.1.1 Study area and data sources

The study area covered the territory of China with geographic coordinates between 73.1–135.5° E and 17.7–53.9° N. A total of 262 distribution records of *S. sphenanthera* were retrieved from publicly accessible databases, including the China Virtual Herbarium (CVH, https://www.cvh.ac.cn/; accessed on 08/12/2022) and the China National Knowledge Infrastructure (CNKI, https://www.cnki.net/; accessed on 08/12/2022). All the retrieved records were filtered to remove dubiously unreliable and redundant data using the method of Ye et al. (2020). To reduce errors caused by clustering effects, we retained only one distribution point in a 5×5 km grid using the ENMtools v1.4.1 software (Warren et al., 2010). A final set of 222 species occurrence points were used for modeling analysis (**Figures S1** and **S2**, **Table S1**).

The raw environmental data used in this study were downloaded from network databases, such as CHELSA (https://chelsa-climate.org/), EarthEnv (http://www.earthenv.org/topography/), and ORNL DAAC (https://doi.org/10.3334/ORNLDAAC/1304/). The environmental variables included climate (Karger et al., 2017; Title and Bemmels, 2018), topography/landform (Amatulli et al., 2018), and soil conditions (Pelletier et al., 2016). Some variables were generated by R code provided in the original document.

A total of 47 environmental variables (**Table S2**) were considered for model prediction of the current (1981–2010) and future periods (2041–2070 and 2071–2100), and the same 47 variables were included in the prediction of each period. All the environmental data had a spatial resolution of 2.5 arc-minute. The geographical scope of the modeling analysis covered the territory of China. To limit the geographical scope, we used the Administrative Map of China and the Map of National Border–Territorial Sea as base maps for masking.

Considering the influence of climate scenario selection on model prediction, we selected the following five atmospheric general circulation models for climate data of future decades: GFDL-ESM4, IPSL-CM6A-LR, MPI-ESM1-2-HR, MRI-ESM2-0, and UKESM1-0-LL. Each model corresponded to a combination of three shared socioeconomic pathways (SSPs) and representative concentration pathways (RCPs): SSP1-RCP2.6, SSP3-RCP7, and SSP5-RCP8.5. The climate severity of the three climate scenarios increased from SSP1-RCP2.6 to SSP5-RCP8.5. To reduce the prediction uncertainty caused by a single atmospheric circulation model, the output layers corresponding to the three climate scenarios were arithmetically averaged. Therefore, 31 sets of climate data were used in this study, with a spatial resolution of 2.5 arc-minutes.

Significant environmental variables were selected by the MaxEnt v3.4.1 (Phillips et al., 2017) and ENMTools v1.4.1 software (Warren et al., 2010) to construct the MaxEnt model. Based on the data of species occurrence points and 47 environmental variables, we identified the most prominent bioclimatic factors that influenced the potential distribution of *S. sphenanthera* (Shcheglovitova and Anderson, 2013). First, the Jackknife method was employed to evaluate the importance of each variable. Second, Pearson correlation coefficients were used to evaluate the correlations among different variables. For each pair of significantly correlated variables (|*r*| ≥ 0.8), only one with a greater significant contribution to habitat suitability was retained (Wei et al., 2021; Yang et al., 2021).

#### 2.1.2 Parameter optimization, model construction, and reliability test

The MaxEnt v3.4.1 software (Phillips et al., 2017) was used to construct a MaxEnt model for *S. sphenanthera*. To ensure that the potential distribution of *S. sphenanthera* was close to normal probability, 75% of the data were selected for model training, and the remaining data for model testing. The maximum number of iterations was 5000, where each process was repeated 20 times. Other parameter settings were set by default (Sun et al., 2020; Yan et al., 2020).

The ‘kuenm’ package in the R v3.4.1 software (Cobos et al., 2019) was used to optimize the feature class (FC) and regularization multiplier (RM) of the MaxEnt model. The RM was initially set to 0.1–4.0 at 0.1 intervals, resulting in 40 values. Then, four FCs (linear, L; quadratic, Q; hinge, H; and product, P) were arranged and combined to create 15 FC combinations (L, P, Q, H, LP, LQ, LH, LPQ, LPH, LQH, LPQH, PQH, PQ, PH, and QH). Thus, 600 parameter combinations were obtained with FC and RM. Based on the optimization results, we selected a statistically significant model that had an omission rate < 0.05 and a small-sample corrected delta Akaike’s information criterion ≤ 2 (Akpoti et al., 2020; Arenas-Castro et al., 2020).

The area under the receiver operating characteristic curve (AUC) was calculated to evaluate the accuracy of model prediction. The AUC range is (0,1), and a greater AUC value indicates a higher model reliability of distinguishing between suitable and unsuitable habitats. AUC ≥ 0.9 indicates high accuracy of model prediction (Guo et al., 2019; Liu et al., 2019).

#### 2.1.3 Prediction of potentially suitable areas, low impact areas, and spatial pattern change

The habitat suitability for a given species is generally scored 0–1, and a higher suitability value indicates a better adaptive ability of the species. The maximum test sensitivity plus specificity (MTSPS) threshold is superior to other threshold options for the classification of potentially suitable areas (Tang et al., 2018). Therefore, we used the MTSPS threshold of 0.1463 in this study, and those areas with suitability values below the threshold were considered unsuitable for *S. sphenanthera*. The suitability values greater than the MTSPS threshold were equally divided into three groups, corresponding to poorly suitable areas (0.1463–0.4309), moderately suitable areas (0.4309–0.7154), and highly suitable areas (0.7154–1), respectively (Ye et al., 2018; Wei et al., 2021).

Low impact areas were defined as those areas where climate change had low impact on *S. sphenanthera* growth (Pan et al., 2020). These areas were identified by overlaying the binary prediction maps of potentially suitable areas in different periods and extracting the completely overlapped portions. First, the maps of potentially suitable areas in different periods were superimposed using the DIVA-GIS v7.5 software (http://www.diva-gis.org/). Then, the spatial units with distribution probability greater or lower than the MTSPS threshold were re-defined as suitable and unsuitable areas, respectively. This led to the establishment of the matrices of unsuitable and suitable areas, and the completely overlapped portions in the overlay layer were extracted as low impact areas. We predicted the low impact areas under three climate scenarios in the current (1981–2010) and future periods (2041–2070 and 2071–2100).

The spatial pattern change of potentially suitable areas across different periods was analyzed using the DIVA-GIS v7.5 software (Zurell et al., 2020; Santos-Hernandez et al., 2021; Wu et al., 2021). Based on the matrix table of suitable and unsuitable areas, we analyzed changes in the percentage of potentially suitable areas for *S. sphenanthera* under current and future climatic conditions.

### 2.2 Determination of secondary metabolites

#### 2.2.1 Sample collection

A total of 32 fresh fruit samples were collected from different SSF-producing regions (cities or counties) throughout China in August 2020 (**Table 1**). The samples were oven-dried at 40°C to constant weight, crushed into powder, and passed through a 200-mesh sieve before analysis.

**Table 1:** Location of the 32 sampling sites in China.

No.	Region	Longitude/E	Latitude/N	No.	Region	Longitude/E	Latitude/N
S1	Huxian County, Shaanxi Province	108.5879	33.7896	S17	Xiangyang City, Hubei Province	111.2831	31.895
S2	Zhen’an County, Shaanxi Province	109.1385	33.4391	S18	Lusi County, Henan Province	111.033	34.008
S3	Huxian County, Shaanxi Province	108.5645	34.1243	S19	Ningshan County, Shaanxi Province	108.3313	33.3628
S4	Tongguan County, Shaanxi Province	110.2707	34.4922	S20	Qingyang County, Shaanxi Province	106.0669	33.234
S5	Tianshui City, Gansu Province	105.7554	34.5706	S21	Diqing Tibetan Autonomous Prefecture, Yunnan Province	99.7136	27.8592
S6	Xiangyang City, Hubei Province	112.1409	32.0103	S22	Chengkou County, Chongqing Municipality	109.1829	31.7847
S7	Nanjiang County, Sichuan Province	106.8614	32.3718	S23	Zhashui County, Shaanxi Province	109.1355	33.6501
S8	Yang County, Shaanxi Province	107.5024	33.2039	S24	Liuba County, Shaanxi Province	106.9495	33.5391
S9	Shiquan County, Shaanxi Province	108.1947	33.208	S25	Tongguan County, Shaanxi Province	110.2397	34.4883
S10	Mianyang City, Sichuan Province	104.8342	31.7529	S26	Foping County, Shaanxi Province	107.9724	33.3985
S11	Pingwu County, Sichuan Province	104.5084	32.2522	S27	Shanyang County, Shaanxi Province	109.8823	33.5322
S12	Hanyin County, Shaanxi Province	108.5088	32.8931	S28	Zhen’an County, Shaanxi Province	109.2144	33.4295
S13	Hanyang County, Hubei Province	114.2186	30.5543	S29	Langao County, Shaanxi Province	108.9316	32.1831
S14	Hanyin County, Shaanxi Province	109.1292	33.6663	S30	Shiquan County, Shaanxi Province	108.0722	33.2874
S15	Xixia County, Henan Province	111.4736	33.3073	S31	Chenggu County, Shaanxi Province	107.3871	33.2157
S16	Taibai County, Shaanxi Province	107.4578	34.0482	S32	Zhashui County, Shaanxi Province	109.0867	33.8344

#### 2.2.2 Chromatographic analysis

Lignans were extracted from SSF samples with methanol under ultrasonic irradiation and analyzed by ultra-high-performance liquid chromatography (Huang et al, 2008). We analyzed the extracts on an Ultimate UHPLC PolarRP C18 (100 mm × 2.1 mm, 1.8 mm) column connected to a Waters UPLCH-Class ultra-high-performance liquid chromatography system. There were six replicates for each sample. Nine standard substances (schisandrol A, B; schisantherin A, B; schisandrin A, B, C; gomisin J; and schisanhenol) were purchased from Solarbio Science & Technology Co. Ltd. (Beijing, China). Major peak identification, standard solution preparation, and methodology validation were conducted following the protocol of Wang et al. (2018).

Volatile oils were isolated by ethyl ether extraction and their chemical composition was analyzed by gas chromatography–mass spectrometry (Wang et al., 2017). Nine replicates were prepared for each sample.

Polysaccharide extraction was performed in ethyl alcohol, and the absorbance of sample extracts was measured by spectrophotometry at 490 nm with glucose as the substrate (Wang et al., 2011). The polysaccharide content in the samples was calculated as follows: polysaccharide content (%) = C*D*F/*W* × 100, where *C* is the concentration of glucose in the test solution (mg/mL), *D* is the dilution factor of the test solution, *F* is the conversion factor (*F* = *w*/*c* × *d*, where *w* is the polysaccharide content (mg), *c* is the glucose concentration in the polysaccharide diluent (mg/mL), and *d* is the dilution factor of polysaccharide), and *W* is the weight of the test sample (mg). Three replicates of each sample were tested.

### 2.3 Data analysis

All tests, including the prediction of potentially suitable habitats and the determination of SM contents, were carried out at least three times. The data were statistically analyzed using the R v4.0.5 software with different packages (yyeasy 0.0.4.0, https://gitee.com/yanpd01/yyeasy; psych 2.1.9, https://CRAN.R-project.org/package=psych; ggplot2 3.3.5, https://CRAN.R-project.org/package=ggplot2) (Robinson et al., 2010; Ginestet, 2011; Love et al., 2014). One-way analysis of variance was conducted to compare group means, and if differences existed, Tukey’s test was performed to assess their significance (*p* < 0.05). Pearson’s correlation analysis and mapping between the 47 environmental variables and the contents of major SMs in SSF were conducted. Pearson’s correlation analysis and mapping were performed among 47 environmental variables. Heatmaps of major SMs in SSF were created using the TBtools v1.113 software (Chen et al., 2020).

## 3 Results

### 3.1 Model parameter optimization and accuracy evaluation

Among the 47 environmental variables, eight were ultimately selected and used to construct the MaxEnt model (**Table 2**). These variables were average soil and sedimentary deposit thicknesses across upland hillslopes and valley bottoms (AvgSoilSedimDeposThick), isothermality (BIO03), annual range of temperature (BIO07), elevation, maximum temperature of the coldest month (maxTempColdest), net primary productivity (NPP), monthly variability in potential evapotranspiration (PETseasonality), and vector ruggedness measure (VRM). NPP (26.19%) was the largest contributor to habitat suitability, followed by BIO07 (18.22%), BIO03 (17.03%), and AvgSoilSedimDeposThick (13.66%). The relative contributions of PETseasonality (9.86%), elevation (8.33%), maxTempColdest (3.75%), and VRM (2.96%) were relatively low (**Figures S3** and **S4**).

**Table 2:** Environmental variables selected for model construction and their contributions to habitat suitability.

Variable	Explanation	Relative contribution (%)
AvgSoilSedimDeposThick	Average soil and sedimentary deposit thicknesses across upland hillslopes and valley bottoms (m)	13.66
BIO03	Isothermality (°C/10)	17.03
BIO07	Annual range of temperature (°C/10)	18.22
Elevation	Elevation (m)	8.33
maxTempColdest	Maximum temperature of the coldest month (°C*10)	3.75
NPP	Net primary productivity (g·C·m^–2^·y^–1^·10^–1^)	26.19
PETseasonality	Monthly variability in potential evapotranspiration (mm/month)	9.86
VRM	Vector ruggedness measure (dimensionless)	2.96

Based on the results of model optimization, the optimal FC and RM were LQ and 0.2, respectively. The AUC_TRAIN_ and AUC_TEST_ values of the optimized model were 0.9630 ± 0.0021 and 0.9615 ± 0.0085, respectively, with an |AUC_DIFF_| value of 0.0015. The partial AUC value was 0.9415 ± 0.0081 (**Figure S5**). All these estimates were indicative of high prediction accuracy.

Based on the MTSPS threshold (0.1463), the spatial units were subdivided as follows: 0-0.1463 Unsuitable areas; 0.1218-0.4309 Poorly suitable areas; 0.4309-0.7069 Moderately suitable areas; 0.7154-1 Highly suitable areas.

### 3.2 Projection of potentially suitable areas and their spatial pattern change

**Figure 1:**
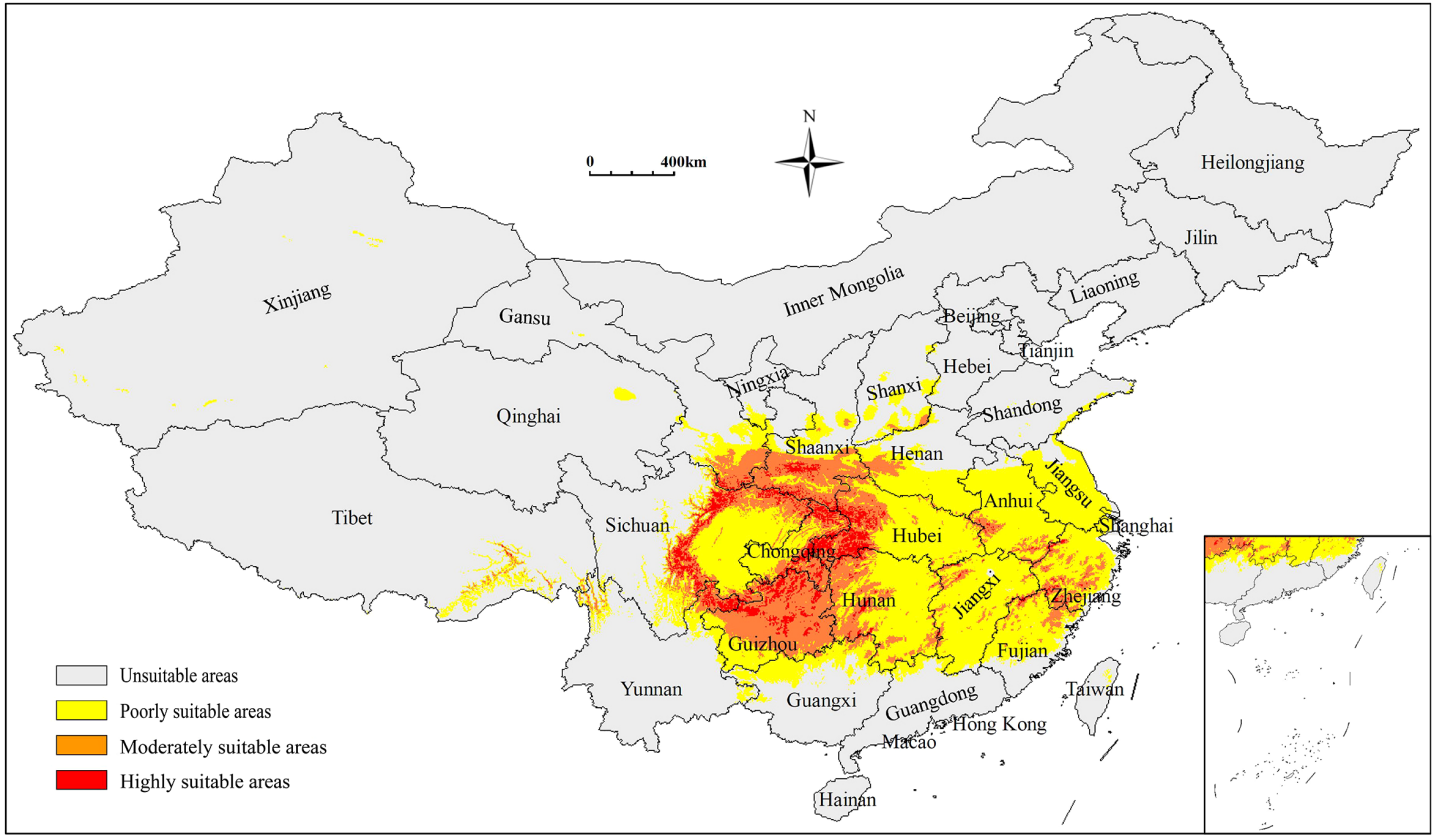
Predicted distribution of *S. sphenanthera* in China under current climatic conditions (1981–2010). 0-0.1463 Unsuitable areas, represented by grey; 0.1218-0.4309 Poorly suitable areas, represented by yellow; 0.4309-0.7069 Moderately suitable areas, represented by orange; 0.7154-1 Highly suitable areas, represented by red.

The potentially suitable areas for *S. sphenanthera* were 179.58×10^4^ km^2^ in total under the current climatic conditions, accounting for 18.71% of China’s territory. These areas were primarily distributed across Shaanxi, Chongqing, Guizhou, Sichuan, and Hubei Provinces (or Municipality; **Figure 1**). Poorly, moderately, and highly suitable areas were 119.00×10^4^ km^2^, 49.61×10^4^ km^2^, and 10.98×10^4^ km^2^, respectively. The highly suitable areas were mainly located in southern Shaanxi, northern Guizhou, southeastern Chongqing, western Hubei, and northern Hunan Provinces (or Municipality; **Table 3**).

**Table 3:** Predicted suitable areas for *S. sphenanthera* in China under current and future climatic conditions.

Climatic scenario and prediction period		Predicted suitable areas (×10^4^ km^2^) and their proportions in current suitable areas			
		Total suitable areas	Poorly suitable areas	Moderately suitable areas	Highly suitable areas
1981–2010		179.58	119.00	49.61	10.98
SSP1-RCP2.6	2041–2070	55.29	54.30	0.98	0.00
		(30.79%)	(45.63%)	(1.98%)	(0.00%)
	2071–2100	52.51	51.84	0.67	0.00
		(29.24%)	(43.56%)	(1.36%)	(0.00%)
SSP3-RCP7	2041–2070	56.50	55.48	1.02	0.00
		(31.46%)	(46.62%)	(2.06%)	(0.00%)
	2071–2100	51.20	50.52	0.68	0.00
		(28.51%)	(42.45%)	(1.37%)	(0.00%)
SSP5-RCP8.5	2041–2070	49.66	48.98	0.67	0.00
		(27.65%)	(41.16%)	(1.36%)	(0.00%)
	2071–2100	43.57	43.50	0.07	0.00
		(24.26%)	(36.56%)	(0.14%)	(0.00%)

**Figure 2:**
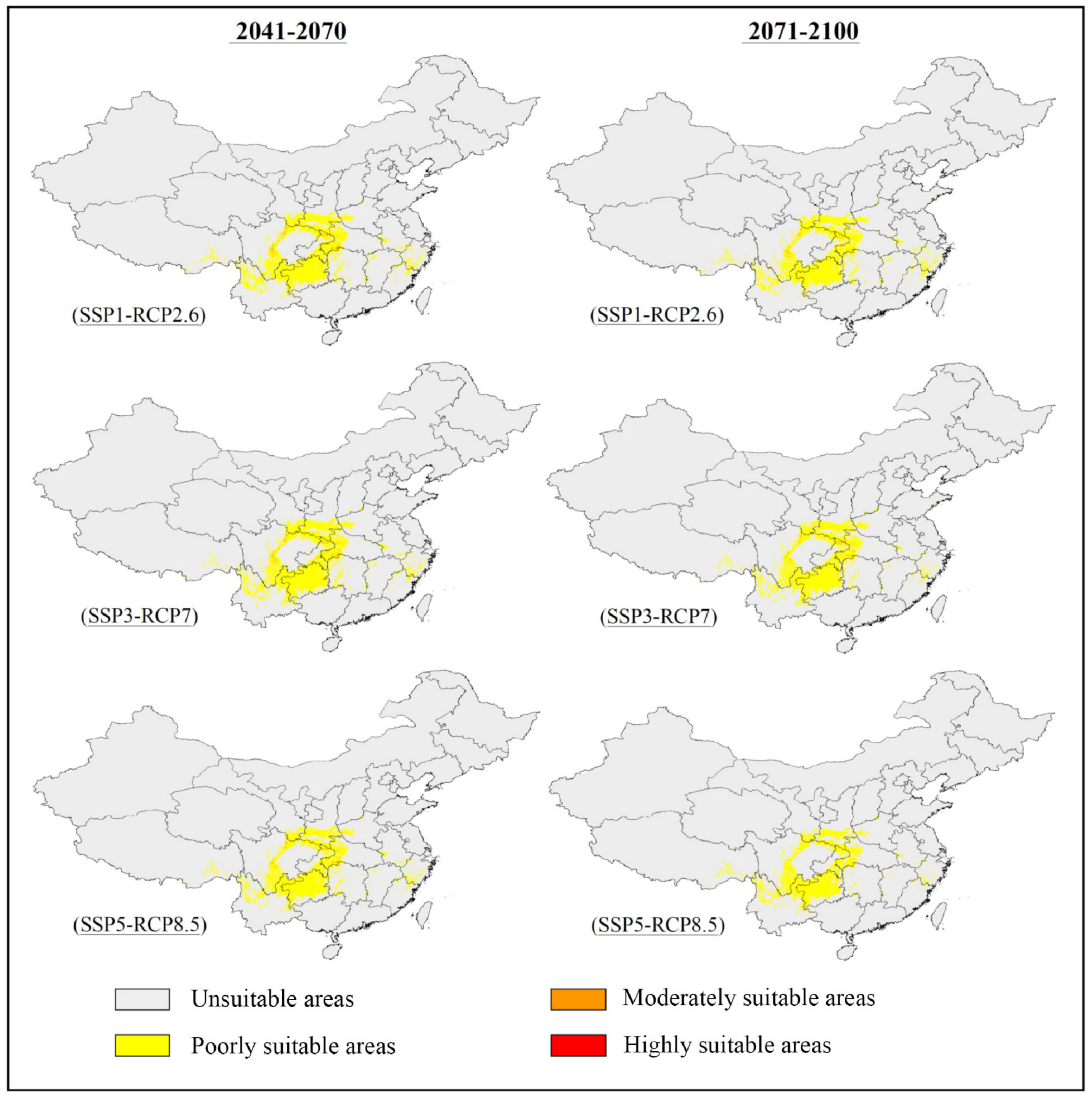
Predicted distribution of *S. sphenanthera* in China under future climatic scenarios (2041–2100).

**Figure 3:**
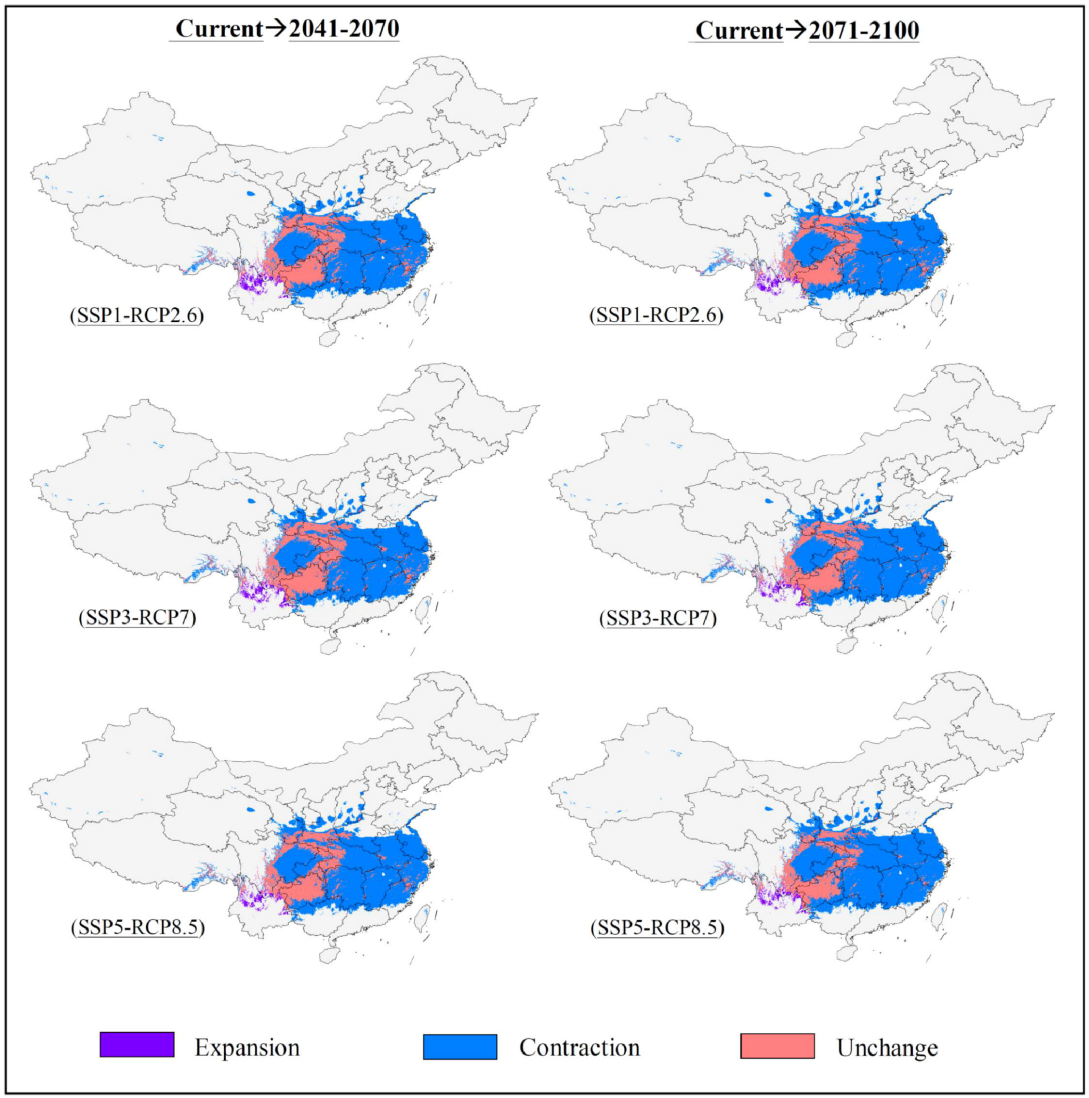
Spatial change of potentially suitable areas for *S. sphenanthera* from the current to future periods.

The potentially suitable areas for *S. sphenanthera* were predicted to decrease from the current to the future, and this trend would be more prominent with increasing severity of climate change (SSP1-RCP2.6 → SSP5-RCP8.5) (**Figures 2**, **3**; **Table 3**). Specifically, the model prediction revealed a reduction in the suitable areas from 1981–2010 to 2071–2100 under the SSP1-RCP2.6 scenario, involving southern Shaanxi, northern Guizhou, southeastern Chongqing, and western Hubei Provinces (or Municipality). Under the SSP3-RCP7 scenario, the total suitable areas would shrink to varying degrees across the two future periods, accounting for 31.46% (2041–2070) and 28.51% (2071–2100) of the current suitable areas. Under the SSP5-RCP8.5 scenario, the future suitable areas would be remarkably smaller than the current suitable areas. The potentially suitable areas would shift westward from the current to the future, and the highly suitable areas would disappear completely by 2100 under different climate scenarios.

### 3.3 Identification of low impact areas

**Figure 4:**
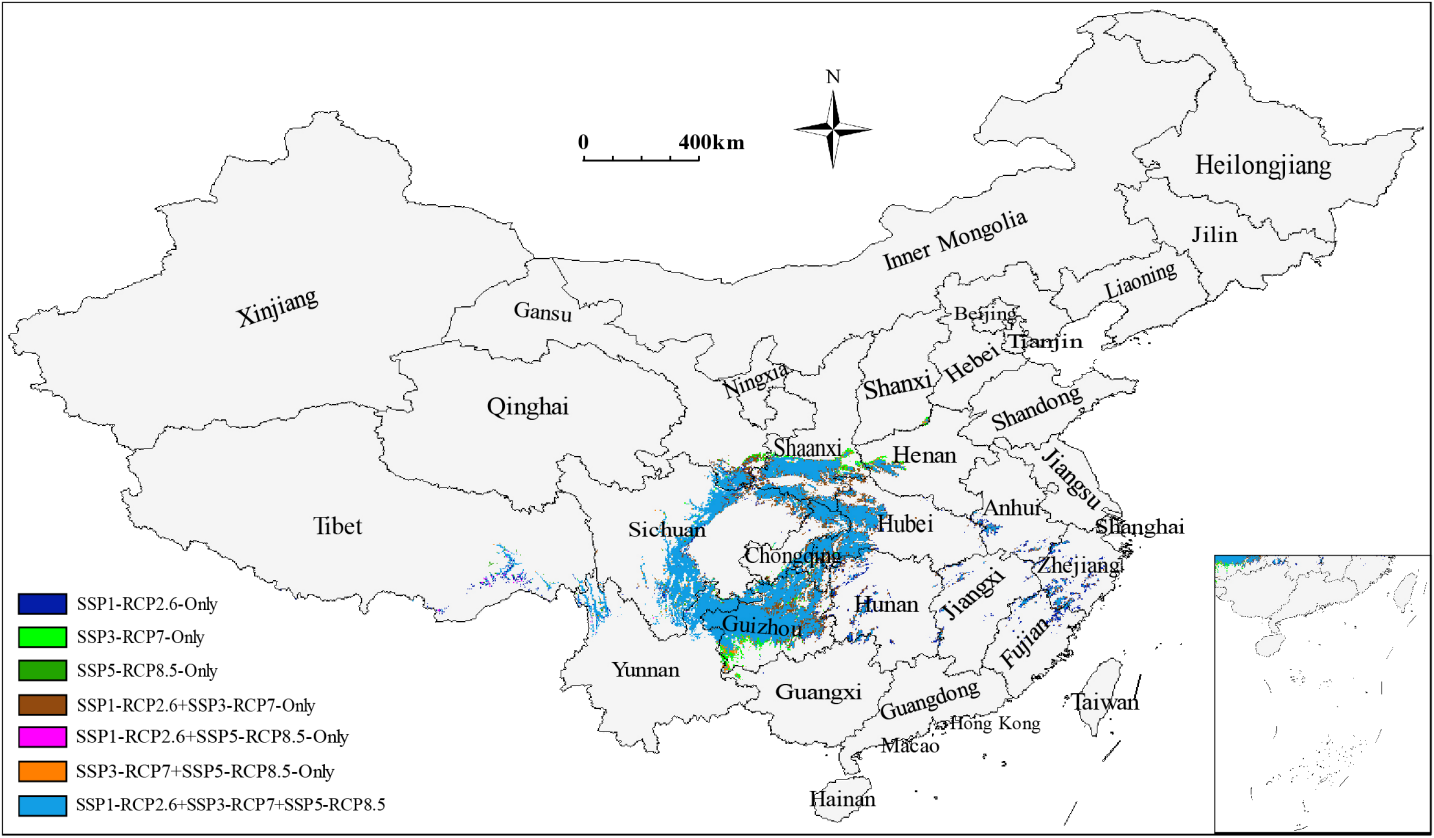
Composite prediction of low impact areas for *S. sphenanthera* under various combinations of climate scenarios.

The total low impact areas for *S. sphenanthera* would decrease from 39.57×10^4^ to 30.49×10^4^ km^2^ as a consequence of increased severity of climate change (SSP1-RCP2.6 → SSP5-RCP8.5). The corresponding proportions of low impact areas in the current suitable areas would also change from 22.03% to 16.98% (**Table 4**). However, specific areas represented by southern Shaanxi, northwestern Guizhou, southeastern Chongqing, and western Hubei Provinces (or Municipality) were identified as stable suitable areas despite different climate scenarios (**Figure 4**). Furthermore, the low impact areas under various combinations of climate scenarios were predicted. The largest low impact areas were obtained under the SSP1-RCP2.6 scenario, accounting for 22.03% of the current suitable areas. The low impact areas were smallest under the SSP1-RCP2.6 + SSP3-RCP7 + SSP5-RCP8.5 scenario combination, accounting for 16.51% of the current suitable areas (**Figures 4** and **S6**).

**Table 4:** Low impact areas for *S. sphenanthera* under different climate scenarios.

Low impact areas	Climate scenario						
	SSP1-RCP2.6	SSP3- RCP 7.0	SSP5- RCP 8.5	SSP1-RCP2.6 + SSP3-RCP7	SSP1-RCP2.6 + SSP5-RCP8.5	SSP3-RCP7 + SSP5-RCP8.5	SSP1-RCP2.6 + SSP3-RCP7 + SSP5-RCP8.5
Geographic area (×10^4^ km^2^)	39.57	38.50	30.49	37.31	30.33	30.25	29.65
Proportion in China’s territory (%)	4.12	4.01	3.18	3.89	3.16	3.15	3.09
Proportion in the current suitable areas (%)	22.03	21.44	16.98	20.78	16.89	16.85	16.51

### 3.4 Accumulation patterns of major secondary metabolites across regions

**Figure 5:**
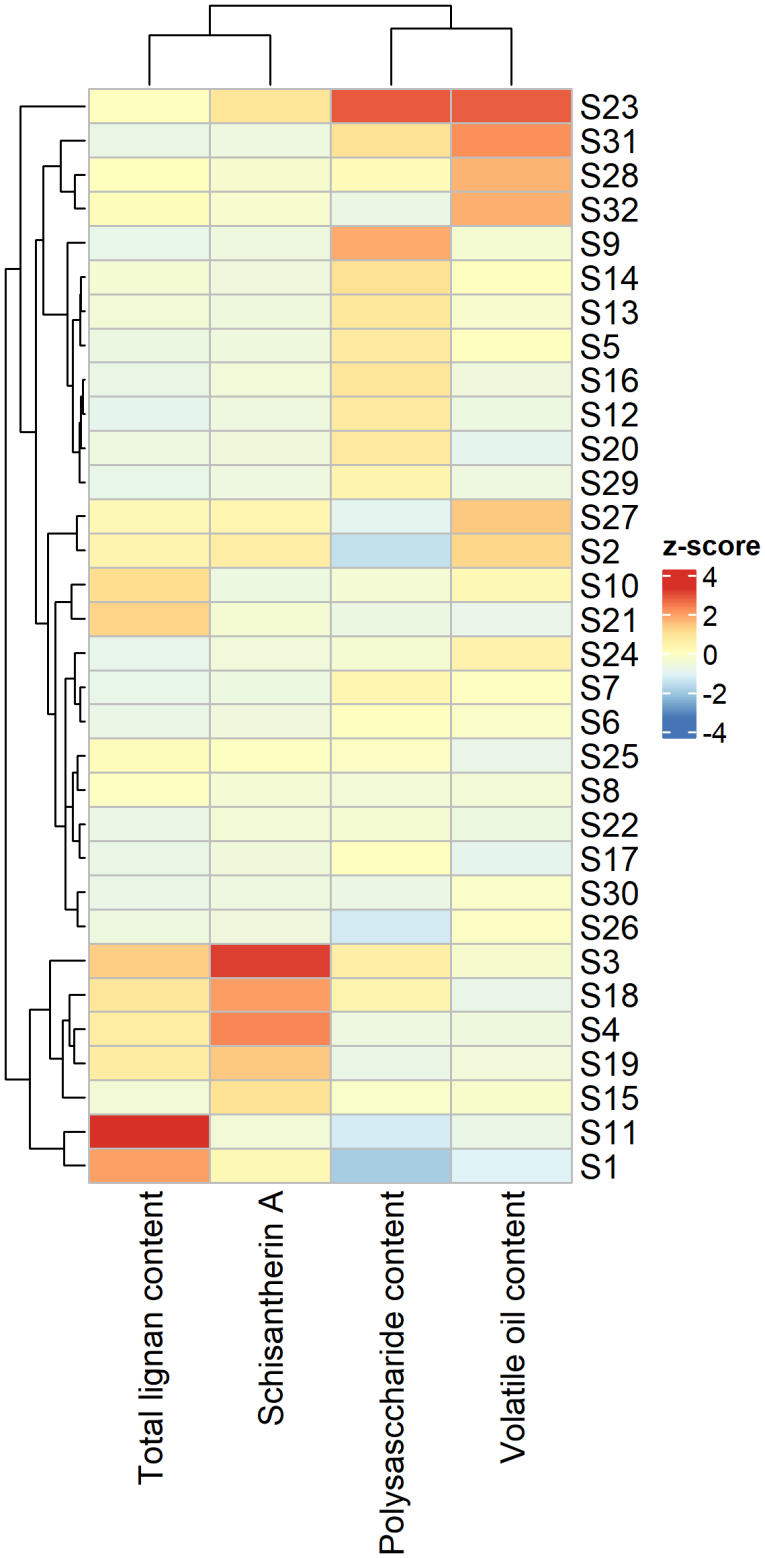
Heatmap showing the contents of major secondary metabolites in Schisandrae Sphenantherae Fructus across 32 regions.

The lignan content of SSF samples varied across different producing regions. Among the nine lignan components identified, schisantherin A was detected at the highest level in all regions, with gomisin J and schizandrol B at the lowest levels (**Figure S7**). The content of schisantherin A was exceptionally high at sites S25, S26, S28, and S32. The highest content of schisanhenol was observed at sites S13, S20, and S21. The volatile oil content of SSF samples also varied distinctly across regions and occurred at the highest levels at sites S31 and S32 (**Figure S8**). Although the composition of SSF was dominated by terpenoids (**Table S3**), there was considerable variation in the contents of volatile oil components (**Figure S9**). The polysaccharide content reached its highest level at sites S23 and S29, in contrast to the lowest levels at sites S1 and S2 (**Figure S10**). The accumulation of major SMs in SSF samples showed distinct patterns in various regions (**Figure 5**). The content of schisantherin A was highest at site S3, whereas the total content of lignans peaked at site S11. The total contents of both volatile oils and polysaccharides reached their maximum levels at site S23 (**Table S4**).

### 3.5 Relationship between metabolite accumulation and environmental factors

**Figure 6:**
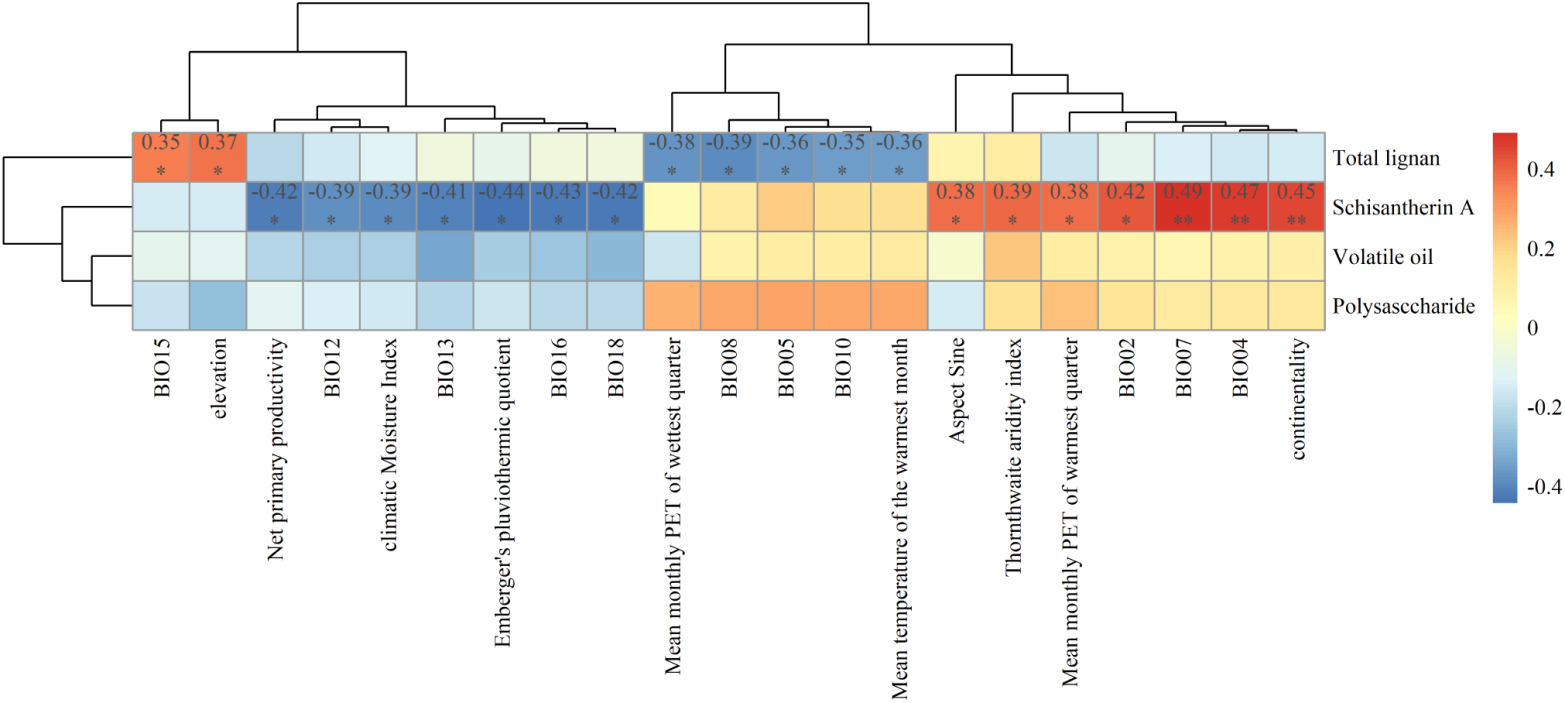
Correlation heatmap between the contents of major secondary metabolites in Schisandrae Sphenantherae Fructus and a subset of environmental variables. * *p* < 0.05 and ** *p* < 0.01.

The content of schisantherin A in SSF showed high correlations with 14 environmental variables (**Figure 6**). For example, it was positively correlated with BIO07, temperature seasonality (BIO04), and mean temperature difference between the hottest and coldest months (continentality) (*p* < 0.01), and there was also a high positive correlation between these environmental factors (**Figure S12**). Conversely, negative correlation was observed between the content of schisantherin A and NPP, mean monthly precipitation of the wettest quarter (BIO16), and mean monthly precipitation of the warmest quarter (BIO18) (*p* < 0.05), and there is a high correlation between these environmental factors (**Figure S12**). The content of schisandrin A was positively correlated with BIO07 and BIO04 (*p* < 0.01). Furthermore, the content of total lignans was positively correlated with precipitation seasonality (BIO15) and elevation (*p* < 0.05), negatively correlated with mean daily temperature of the wettest quarter (BIO08) and mean monthly potential evapotranspiration of the wettest quarter (PETWettestQuarter) (*p* < 0.05). We also found that there was a high positive correlation between the environmental variables that had the same effect on the accumulation of the SMs of SSF, and the environment variables that had the opposite effect on the accumulation of SMs of SSF were negatively correlated (**Figure S11**). The correlations between major metabolite contents and the 47 environmental variables are shown in **Figure S12**.

## 4 Discussion

### 4.1 Comprehensiveness and rationality of MaxEnt model prediction

MaxEnt modeling has become a popular tool in global change biology and biogeography, especially for predicting the potential distribution of animal and plant species (Araujo et al., 2005; Araujo and Luoto, 2007). Compared with other ENM models, the MaxEnt model uses relatively simple continuous or categorical variables and performs well with limited or incomplete datasets (Elith et al., 2011). However, previous studies have often neglected the optimization of model parameters, or the proposed models usually require further optimization, which may affect the prediction accuracy (Garcia-Valdes et al., 2013; Li et al., 2020a; Yan et al., 2020). Therefore, we developed an optimized MaxEnt model to predict potentially suitable areas for *S. sphenanthera* in China. The proposed model achieved high prediction accuracy based on a large AUC value.

In this study, a total of 47 environmental variables were considered in MaxEnt model prediction. The environmental dataset included land cover, soil regolith and sedimentary deposit thickness, cloud cover, topography, temperature, and precipitation. This dataset was more comprehensive than previously used in other studies. Many ENM-based studies have developed prediction models only using environmental data layers from WorldClim (Aidoo et al., 2023; Akyol et al., 2023). For example, Parveen et al. (2022) selected 11 temperature and eight precipitation-derived variables to build a MaxEnt model, and then used it to predict the distribution of potentially suitable habitats for the genus *Nymphaea* in India.

Furthermore, we took into consideration various factors affecting plant growth to optimize the MaxEnt model parameters systematically. These factors included topography, land cover, and cloud cover (Tuanmu and Jetz, 2014; Wilson and Jetz, 2016; Amatulli et al., 2018). The MaxEnt model was run for a maximum of 5000 iterations, with each process repeating 20 times, which could reduce prediction errors. Collectively, our analysis procedures ensured the accuracy, comprehensiveness, and rationality of the optimized MaxEnt model.

### 4.2 Current and future suitable areas for *S. sphenanthera*

Based on MaxEnt modeling, the current suitable areas for *S. sphenanthera* are mainly located in southern Shaanxi, northern Guizhou, southeastern Chongqing, western Hubei, and northern Hunan Provinces, in agreement with previous studies (Heyu et al., 2016). Temperature, precipitation, and soil conditions are major environmental factors driving medicinal plant distribution (Li et al., 2020b). However, the primary factors that determine the range of potentially suitable areas for diverse medicinal plants are variable. For example, Hu et al. (2012) identified mean annual and extreme temperature (minimum and maximum) as the most important limiting factors for *Schisandra chinensis* (Turcz.) Baill during the critical growth period. This is inconsistent with our finding that the growth of *S. sphenanthera* is principally influenced by the carbon sequestration capacity of plants (NPP), temperature-dependent climatic factors (BIO07 and BIO03), and soil physical quality (AvgSoilSedimDeposThick).

NPP is primarily determined by photosynthetic energy and mainly constrained by light, water, and nutrient resources, which are necessary for plant yield formation (Briggs et al., 2018). Temperature and soil physical conditions are also essential factors for plant growth. Field surveys and research reports indicate that *S. sphenanthera* prefers to grow in warm and humid climate zones with a long, sunny, and warm summer (Zhang et al., 2014). Specifically, the mean annual temperature of 15–20°C, the mean annual precipitation of 1,000–2,000 mm, and the soil thickness of 30 cm are favorable conditions for *S. sphenanthera* growth (Lu and Chen, 2009). These exciting findings corroborate the accuracy of the MaxEnt model proposed in this study.

There will be a remarkable trend of decreasing suitable areas for *S. sphenanthera* in the future decades. Predictions under the SSP5-RCP8.5 scenario produced the worst outcome, followed by SSP3-RCP7 and SSP1-RCP2.6. This means that the wild resources of *S. sphenanthera* in China will be depleted in the 21^st^ century as a consequence of decreased growing areas under possible climate change. Under all three climate scenarios, the potentially suitable areas will shift westward from the current to the future and eventually disappear in specific regions of southeastern China. Various plant species respond differently to climate change in terms of geographical distribution. For example, the suitable habitat distribution of *Paeonia veitchii* was predicted to shift toward higher elevations and latitudes under the RCP8.5 scenario (Zhang et al., 2019a). A northward shift and an eastward expansion of planting boundaries were predicted for rice and maize cultivars under the SRES A1B and A2 scenarios similar to RCP8.5 (Yuan et al., 2012; Zhang et al., 2017).

### 4.3 Influence of environmental factors on medicinal plant quality

As expected, the habitat environment had a profound influence on the accumulation of SMs in SSF. This was demonstrated by the correlations between the contents of various SMs in 32 samples of SSF collected so far and different environmental factors. Temperature- and precipitation-dependent climatic factors were the major drivers of SM accumulation patterns in SSF. Specifically, the content of schisantherin A exhibited an upward trend in response to increasing annual temperature extremes (i.e., difference between the annual maximum and minimum temperatures) or seasonal temperature variation. The opposite trend was observed for schisantherin A with increasing annual or seasonal precipitation. Additionally, the total content of lignans exhibited an upward trend with increasing seasonal precipitation and elevation, whereas a downward trend emerged with increasing temperature and evapotranspiration of the wettest quarter. All these findings lead us to posit that specific environmental stress could enhance the accumulation of major medicinal components in SSF. A greater change in temperature or precipitation is likely to enable *S. sphenanthera* plants to accumulate more lignans (especially schisantherin A) in the fruit. In the analysis of environmental factors’ autocorrelation, it was found that there is correlation among the environmental factors that affect the content of SMs in SSF. For example, elevation and BIO15 (precipitation seasonality) were positively correlated with the total lignan content of SSF, and there was also a high positive correlation between these two environmental factors. On the other hand, environmental factors such as BIO08 (mean Temperature of wettest quarter) and BIO05 (max temperature of warmest month) were negatively correlated with the total lignan content. However, these environmental factors show a negative correlation with elevation and BIO15 (**Figures 6** and **S11**). Such correlation aligns with the fact that as altitude increases, temperature gradually decreases (Kad et al., 2022). Therefore, when selecting planting areas for high-quality *S. sphenanthera*, it is possible to summarize these environmental factors that affect the content of one medicinal component (such as total lignans) and make a comprehensive decision on the planting areas for *S. sphenanthera*. Within the suitable habitat range, *S. sphenanthera* plants would produce SSF with higher levels of schisantherin A when growing in areas with higher temperature, less intense light, and lower precipitation. More lignans would accumulate in SSF when planting *S. sphenanthera* in low-temperature areas.

In addition to their medicinal functions, SMs play an essential role in plant adaptation to abiotic stresses, including temperature, drought, nutrient deficiency, carbon dioxide elevation, salinity, and ultraviolet light (Renault et al., 2010; Ramakrishna and Ravishankar, 2011; Rao et al., 2021). For example, terpenoids accumulate in plants in response to abiotic stresses, providing antioxidant protection against drought, temperature, light, and salt stresses (Zhang et al., 2019b). Additionally, nitrogen-containing compounds, such as alkaloids and glucosinolates, occur at high levels in plants with stress responses, which can alleviate oxidative stress (Zhu et al., 2015). Our study also found that the accumulation of some SMs in SSF was stimulated under certain environmental stress. Therefore, the selection of planting areas for *S. sphenanthera* should take into account not only the habitat suitability, but also the accumulation of medicinal components. In this study, samples were collected from 32 *S. sphenanthera* growing areas. Results indicate that the content of lignans in Huxian County, Shaanxi Province, and Pingwu County, Sichuan Province, were relatively high, while polysaccharides and volatile oil content were higher in Zhashui County, Shaanxi Province. These locations are also considered moderately to highly suitable for the growth of *S. sphenanthera*. Therefore, priority could be given to the cultivation of *S. sphenanthera* in these areas.

Like environmental factors, human activities play a non-negligible role in shaping the geographical distribution of *S. sphenanthera* (Zhang et al., 2014). In fact, no single environmental and human factors are expected to independently control the quality of medicinal plants. A combination of multiple factors can provide a better explanation for the variation in medicinal plant quality (Shen et al., 2010). This underscores the necessity of taking into account both environmental factors (e.g., climate, soil, terrain) and human activities (e.g., fertilization, irrigation) when planting *S. sphenanthera* for high-yield and high-quality production of SSF.

## 5 Conclusions

This study developed an optimized maximum entropy model and successfully used it to predict the distribution patterns and spatial changes of potentially suitable areas for *S. sphenanthera* in China. We found that the potentially suitable areas would shrink from the current (1981–2010) to the future (2041–2100), depending on the severity of climate change. The areas of low climate impact were mainly found in southern Shaanxi, northern Guizhou, northwestern Guizhou, southeast Chongqing, and western Hubei Provinces. These low impact areas should be prioritized to establish nature reserves for the conservation of wild *S. sphenanthera* resources or develop farmland for the cultivation of this medicinal plant. The contents of secondary metabolites in Schisandrae Sphenantherae Fructus varied across regions as a consequence of different climatic environments. Diurnal temperature variation, together with seasonal and annual precipitation, prominently influenced the accumulation of secondary metabolites as medicinal components and stress response regulators. Our findings indicate that specific environmental stress is conducive to the improvement of medicinal plant quality. This research can provide scientific guidance for the conservation planning of wild plant resources, large-scale cultivation of *S. sphenanthera*, and quality improvement of finished medicinal materials.

## Data availability statement

The original contributions presented in the study are included in the article/**Supplementary Material**. Further inquiries can be directed to the corresponding authors.

## Author contributions

JS: Formal Analysis, Methodology, Visualization, Writing – original draft, Writing – review & editing. QZ: Methodology, Software, Visualization, Writing – review & editing. PY: Methodology, Software, Visualization, Writing – review & editing. MS: Software, Visualization, Writing – review & editing. HS: Project administration, Validation, Writing – review & editing. HL: Conceptualization, Resources, Writing – review & editing. DZ: Funding acquisition, Investigation, Resources, Writing – review & editing. ZQ: Conceptualization, Data curation, Writing – review & editing. LC: Conceptualization, Data curation, Resources, Writing – review & editing.
