# Supplementary material for: Environmental factors influencing potential distribution of Schisandra sphenanthera and its accumulation of medicinal components
Source: Front Plant Sci. 2023 Dec 12;14:1302417. doi: 10.3389/fpls.2023.1302417 (PMC10756911; doi:10.3389/fpls.2023.1302417)
Supplement: Supplementary file 1 [file DataSheet_1.docx]

**Supplementary material for**

**Environmental factors influencing potential distribution of** ***Schisandra sphenanthera*** **and its accumulation of medicinal components**

Jingjing Shang

The Key Laboratory of Medicinal Resources and Natural Pharmaceutical Chemistry, The Ministry of Education, National Engineering Laboratory for Resource Development of Endangered Crude Drugs in Northwest China, College of Life Sciences, Shaanxi Normal University, Xi’an, Shaanxi, China

**Figure S1.** Distribution of *Schisandra sphenanthera* occurrence points in China (*n =* 222)


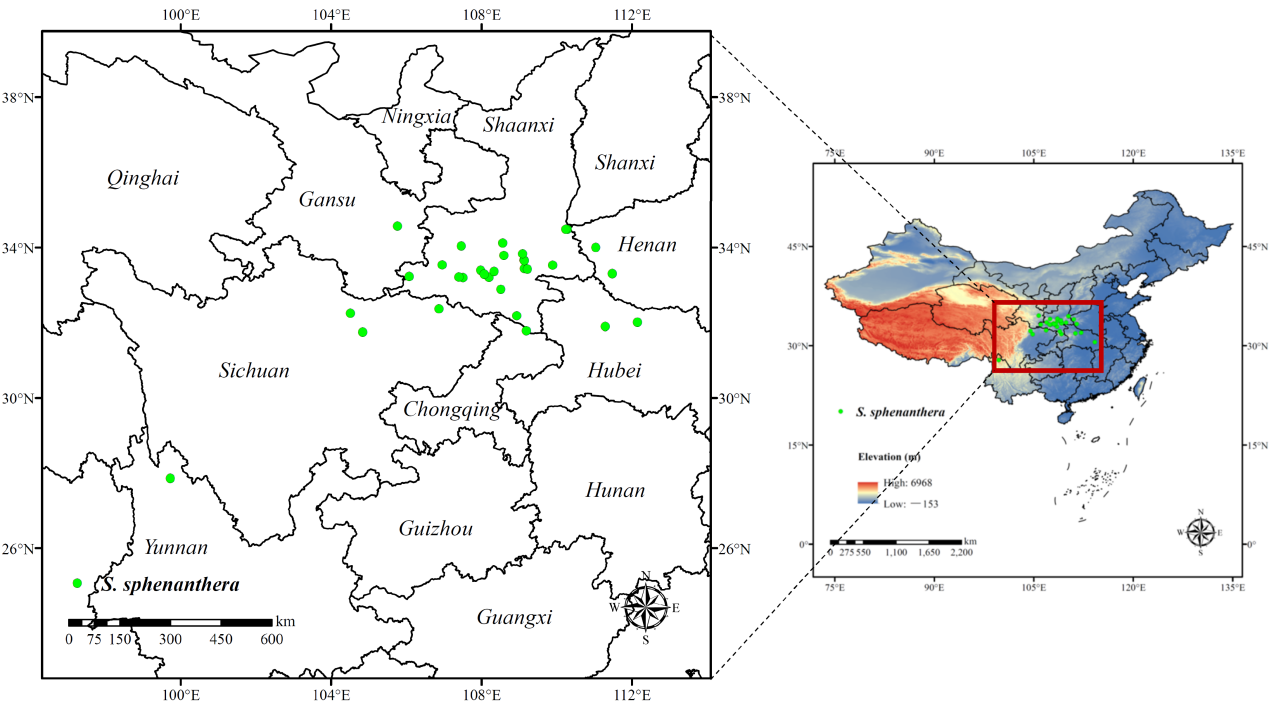


**Figure S2.** Location of *S. sphenanthera* sampling sites across 32 regions in China


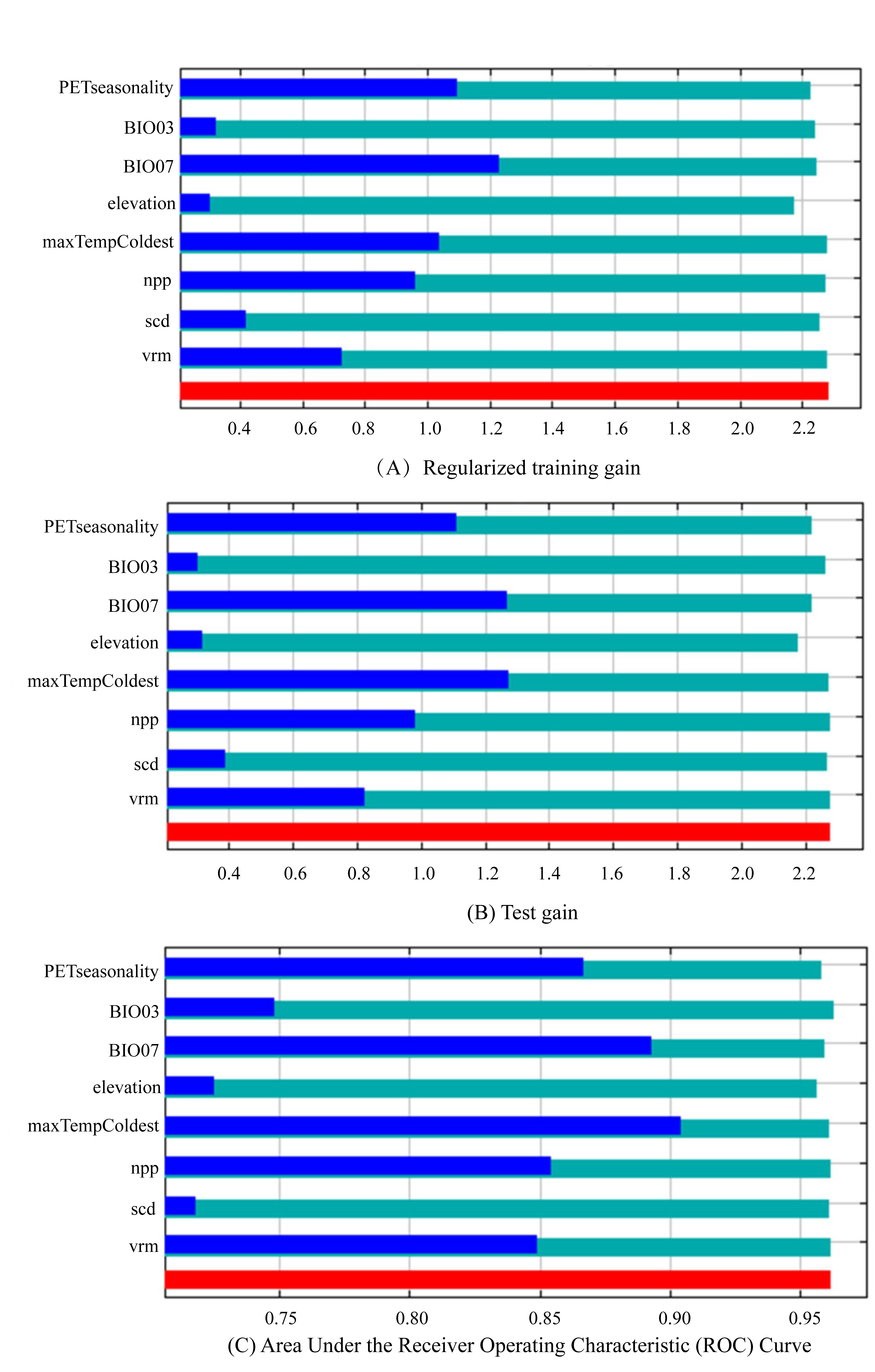


**Figure S3.** Jackknife test for the importance of eight selected environmental variables to habitat suitability for *S. sphenanthera*. (A) Regularized training gain; (B) Test gain; and (C) Area under the receiver operating characteristic curve. Blue, green, and red bars represent the results obtained by running the MaxEnt model with a given variable, without this variable, and with all variables, respectively. PETseasonality, monthly variability in potential evapotranspiration; BIO03, isothermality; BIO07, annual range of temperature; maxTempColdest, maximum temperature of the coldest month; NPP, net primary productivity; SCD, number of snow cover days; and VRM, vector ruggedness measure
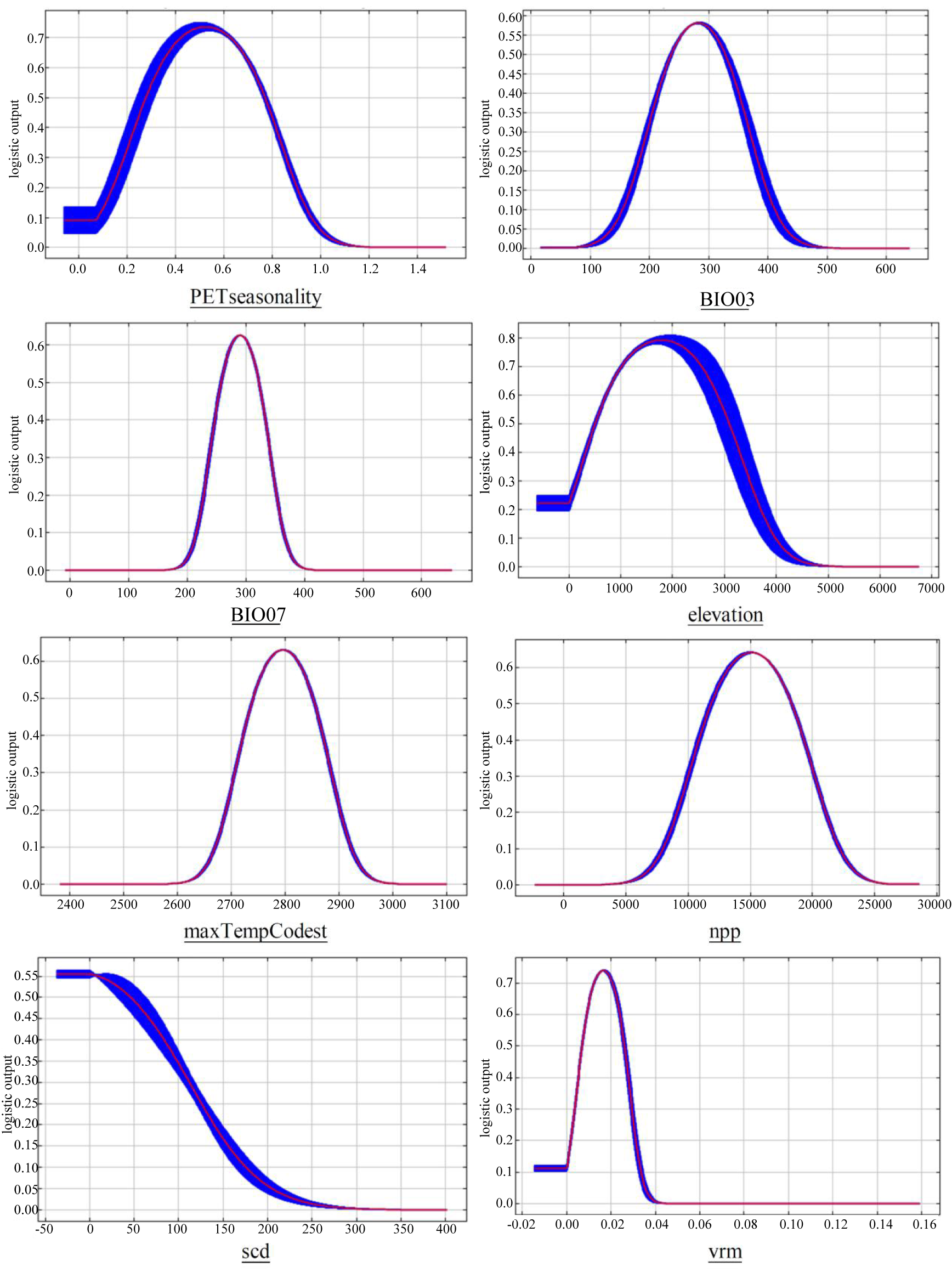


**Figure S4.** Response curves of habitat suitability to the eight environmental variables used for MaxEnt modeling. The plots show the dependence of predicted suitability (y-axis) on the selected variables (x-axis). Parameter abbreviations are defined in Figure S3 legend


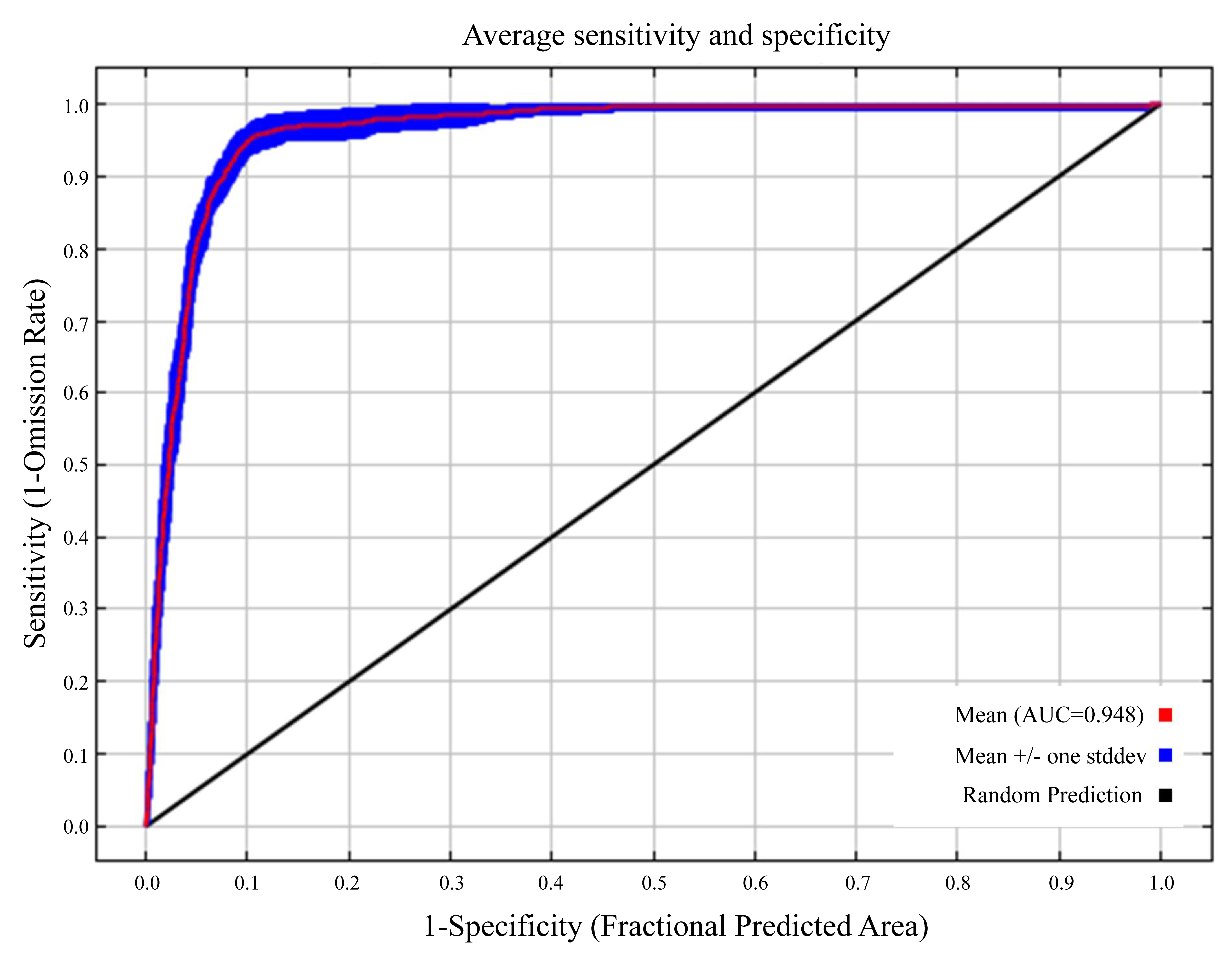


**Figure S5.** The receiver operating characteristic (ROC) curve and the area under ROC curve (AUC) for MaxEnt modeling. The AUC values ranged from 0.90 to 1.00, indicating excellent prediction accuracy


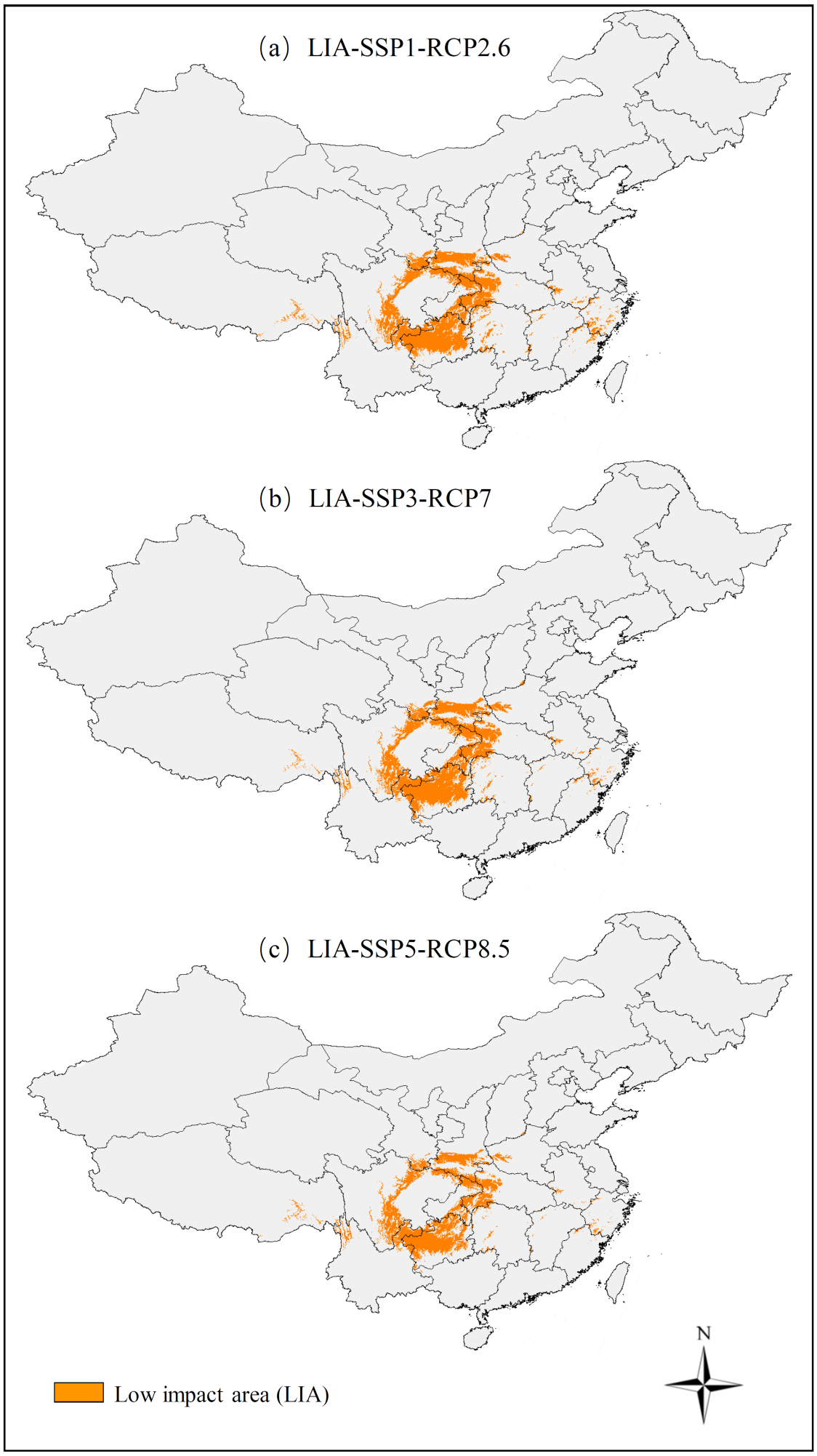
**Figure S6.** Areas of low climate impact for *S. sphenanthera* under three shared socioeconomic pathways (SSP1-RCP2.6, SSP3-RCP7, and SSP5-RCP8.5). The orange color represents the areas with relatively stable and high habitat suitability
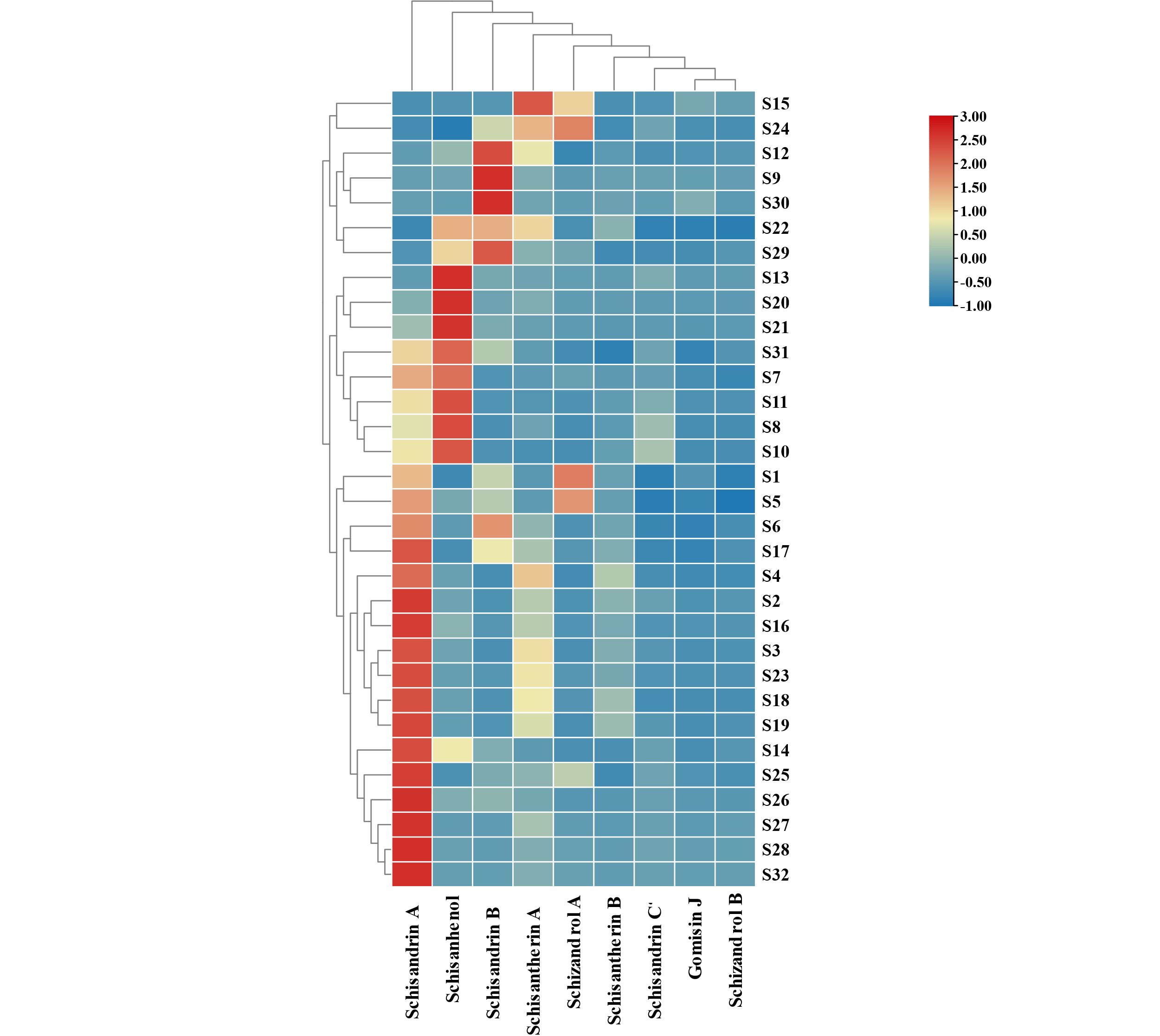


**Figure S7.** Heatmap showing the contents of nine lignin components identified in Schisandrae Sphenantherae Fructus samples collected from 32 regions (S1 to S32)


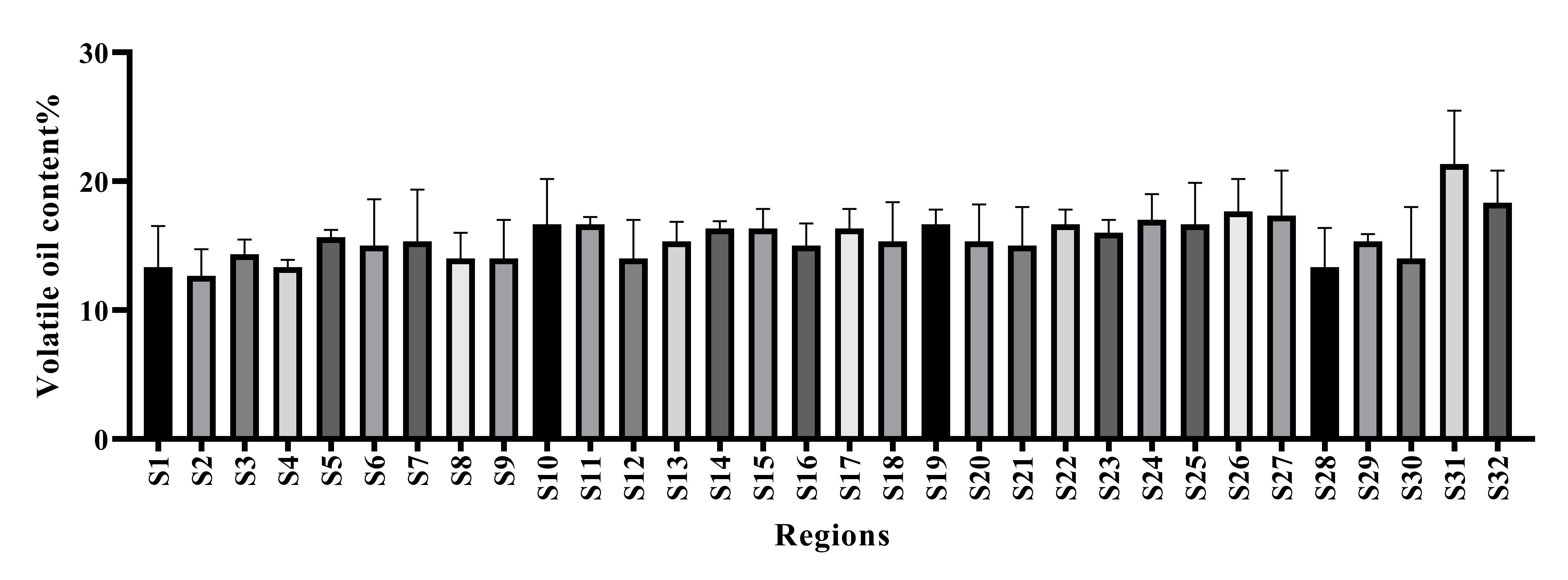


**Figure S8.** Total content of volatile oils in Schisandrae Sphenantherae Fructus across 32 regions. Error bars represent standard error of the mean (*n =* 3).


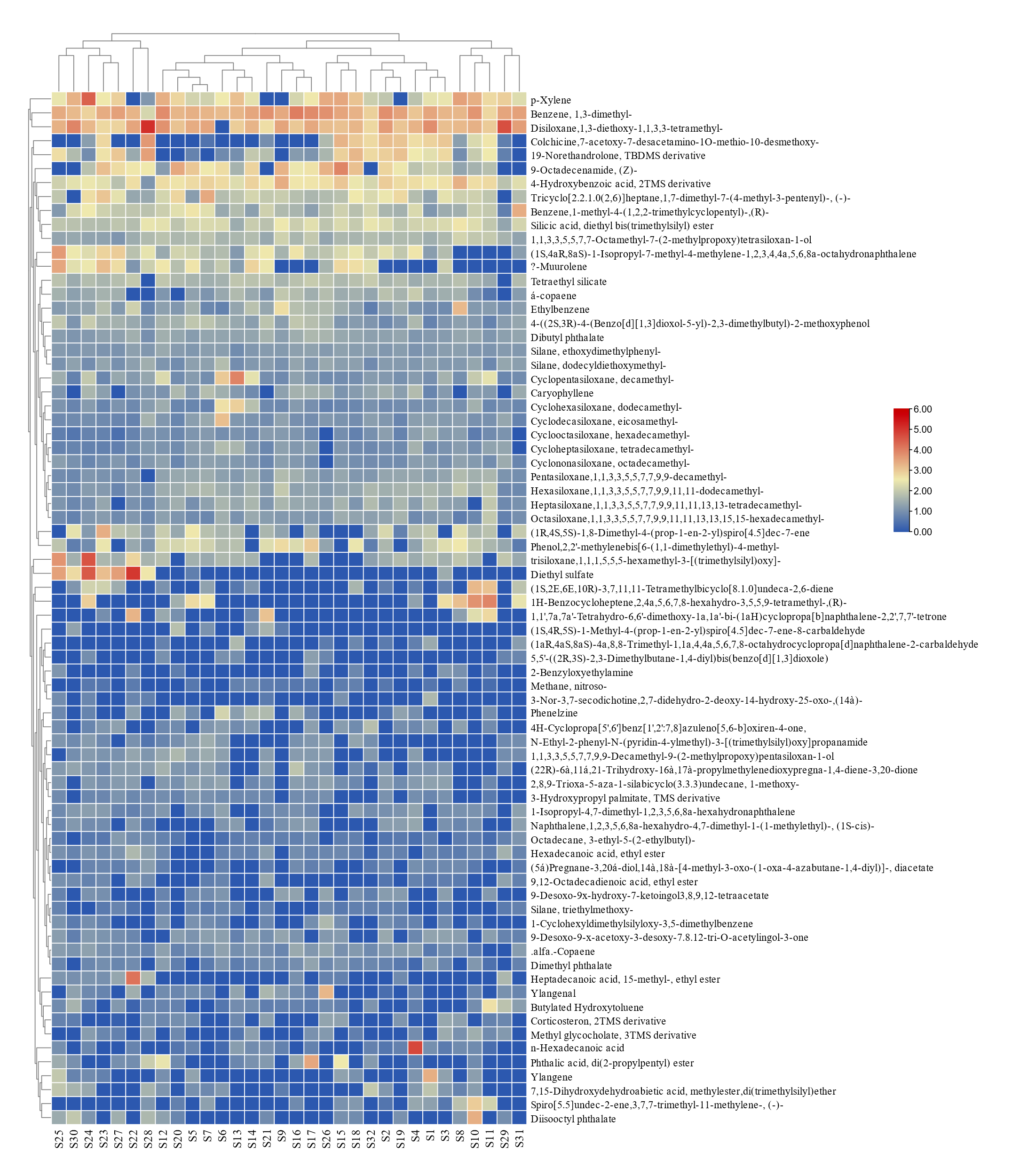


**Figure S9.** Clustering heatmap showing relative contents of medicinal compounds identified in Schisandrae Sphenantherae Fructus across 32 regions


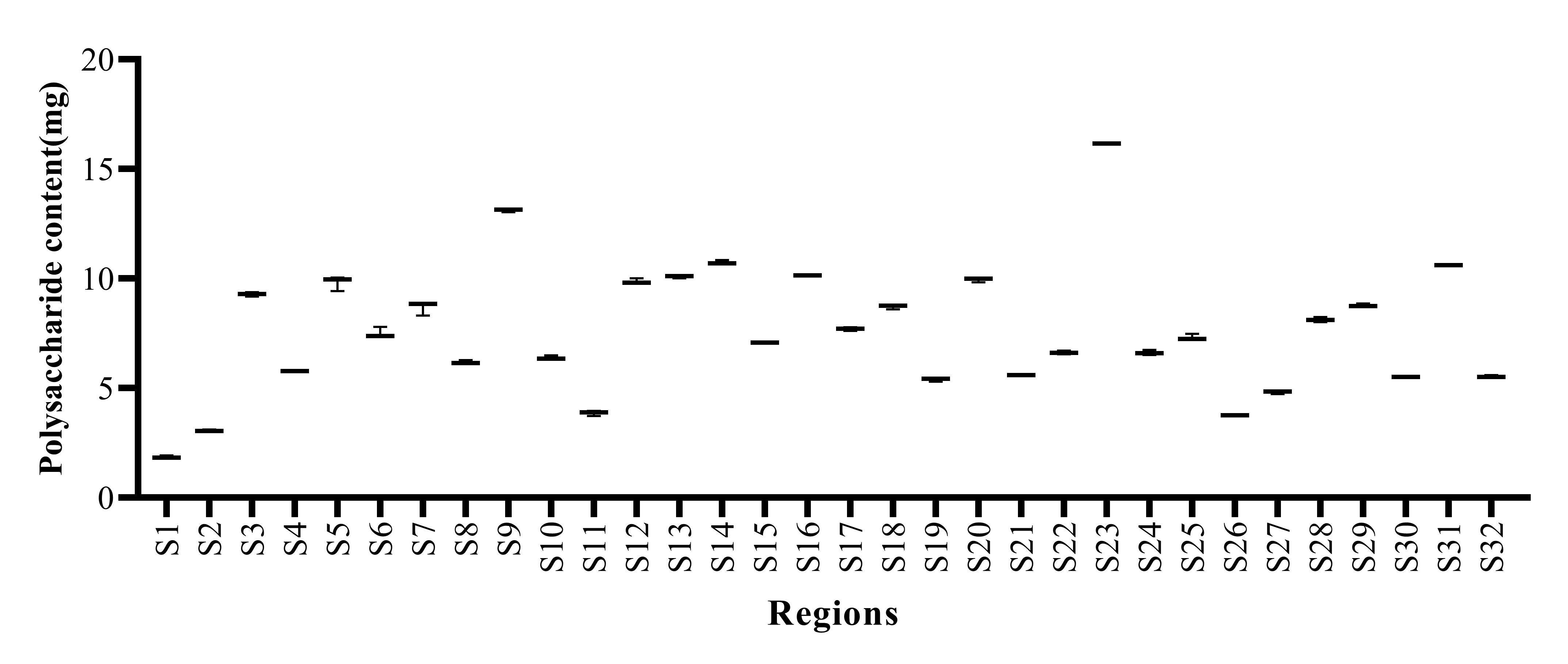


**Figure S10.** Total content of polysaccharides in Schisandrae Sphenantherae Fructus across 32 regions


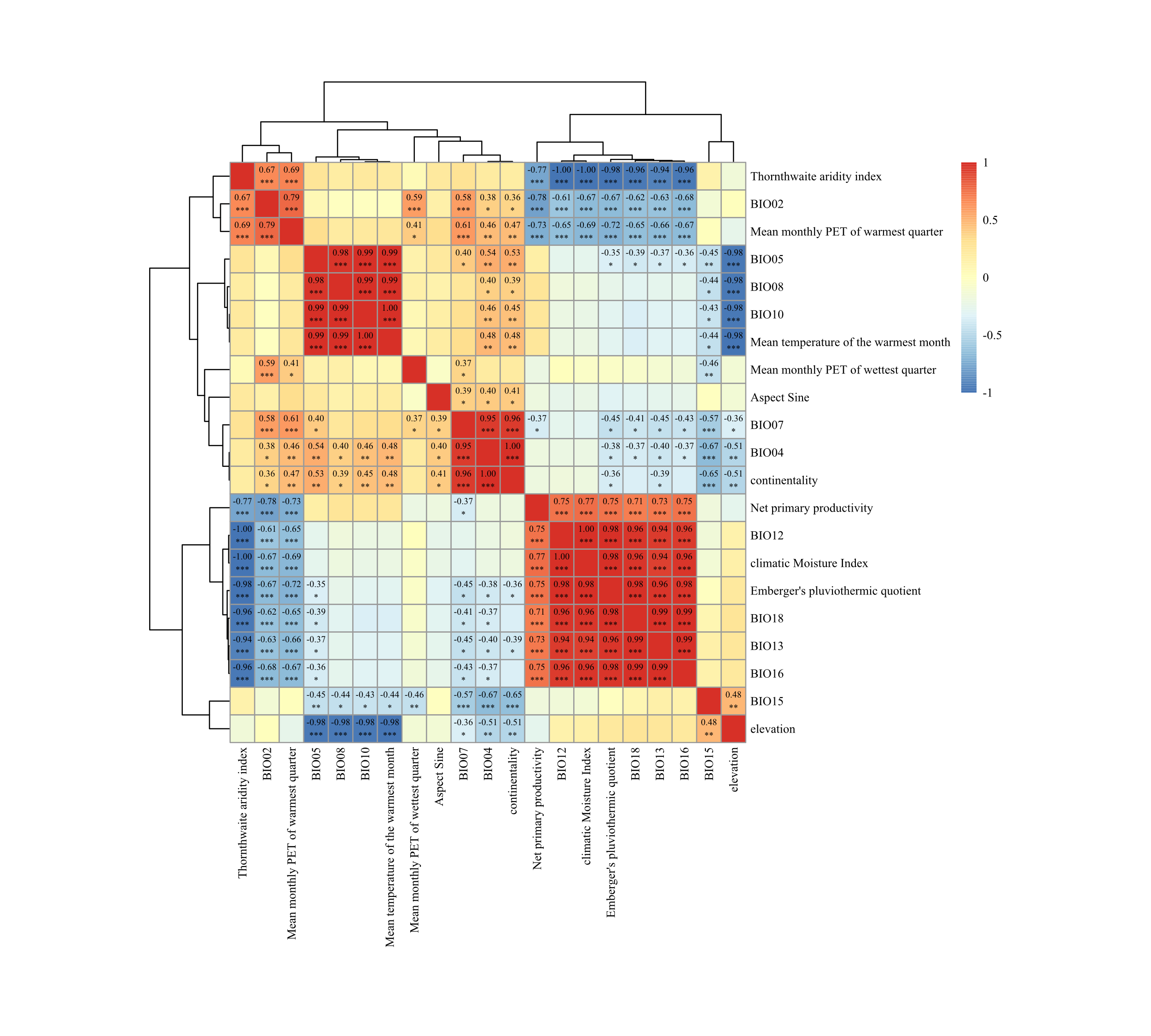


**Figure S11.** Autocorrelation analysis among 21 environmental factors

Note: these environmental factors only included those related to the accumulation of secondary metabolites in Schisandrae Sphenantherae Fructus


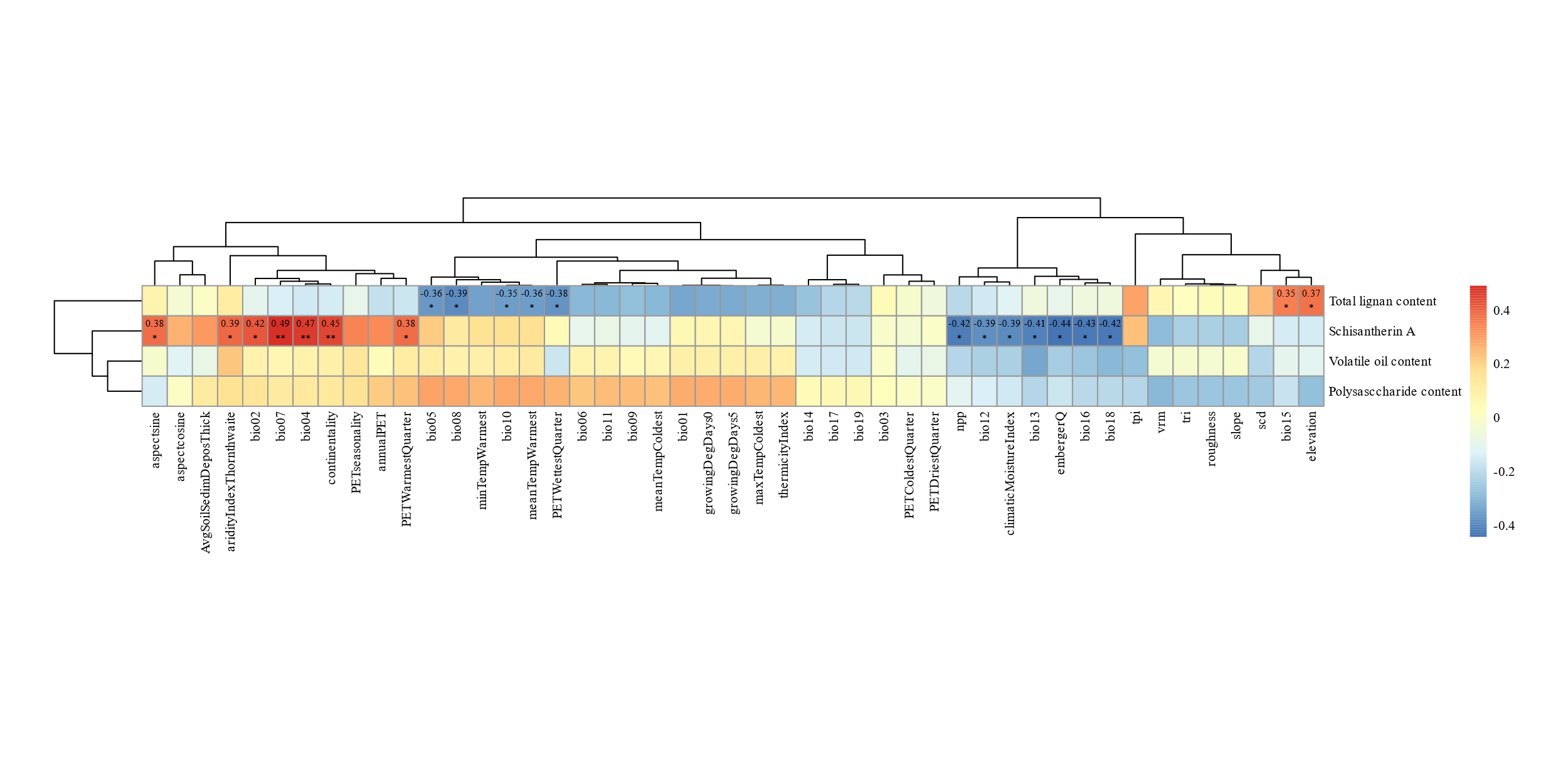
**Figure S12.** Correlation heatmap between the contents of major secondary metabolites in Schisandrae Sphenantherae Fructus and 47 environmental variables used for model prediction. Parameter abbreviations are defined in Table S3. **Table S1.** Rarefied data points of *Schisandra sphenanthera* in China (*n =* 222)

| **Record No.** | **Longitude** | **Latitude** | **Record No.** | **Longitude** | **Latitude** | **Record No.** | **Longitude** | **Latitude** |
| --- | --- | --- | --- | --- | --- | --- | --- | --- |
| 1 | 106.8545 | 34.5825 | 75 | 106.8499 | 34.5801 | 149 | 103.7741 | 31.2642 |
| 2 | 107.7166 | 34.0935 | 76 | 107.7181 | 34.0934 | 150 | 104.7088 | 32.4599 |
| 3 | 107.7111 | 34.0914 | 77 | 107.7176 | 34.0939 | 151 | 103.7299 | 28.2518 |
| 4 | 107.3094 | 34.2664 | 78 | 103.2114 | 30.7710 | 152 | 103.4233 | 30.8452 |
| 5 | 108.7320 | 33.1570 | 79 | 102.9493 | 32.1204 | 153 | 103.555 | 30.9611 |
| 6 | 107.7166 | 34.0935 | 80 | 102.9514 | 30.5401 | 154 | 103.2283 | 30.7835 |
| 7 | 107.7182 | 34.0938 | 81 | 103.5916 | 31.0269 | 155 | 114.1438 | 26.6362 |
| 8 | 107.8217 | 34.0617 | 82 | 104.2884 | 31.6928 | 156 | 115.2409 | 25.2723 |
| 9 | 107.9153 | 34.1389 | 83 | 105.6259 | 31.7264 | 157 | 114.5948 | 28.5582 |
| 10 | 110.6005 | 34.8331 | 84 | 104.8700 | 31.7994 | 158 | 114.0936 | 27.9172 |
| 11 | 108.8517 | 32.0595 | 85 | 105.4923 | 32.5062 | 159 | 115.9685 | 29.5295 |
| 12 | 109.0716 | 32.2879 | 86 | 104.2468 | 33.2668 | 160 | 114.7809 | 28.8617 |
| 13 | 109.3158 | 32.0199 | 87 | 102.7744 | 30.0638 | 161 | 115.9444 | 29.5333 |
| 14 | 108.8517 | 32.0595 | 88 | 105.0836 | 32.4735 | 162 | 115.9746 | 29.4520 |
| 15 | 109.3324 | 31.0235 | 89 | 107.0600 | 32.1821 | 163 | 114.6057 | 28.5397 |
| 16 | 109.4023 | 31.9645 | 90 | 108.3642 | 31.8290 | 164 | 115.7819 | 29.5433 |
| 17 | 108.7099 | 33.2927 | 91 | 107.1996 | 32.3860 | 165 | 114.8665 | 28.9784 |
| 18 | 106.2388 | 32.7805 | 92 | 102.3077 | 29.0043 | 166 | 115.0150 | 28.9388 |
| 19 | 107.9621 | 33.6319 | 93 | 102.6545 | 29.1072 | 167 | 115.9525 | 29.5460 |
| 20 | 108.8583 | 33.7691 | 94 | 104.2629 | 33.1778 | 168 | 115.9716 | 29.4784 |
| 21 | 108.8935 | 33.8027 | 95 | 102.9167 | 28.0877 | 169 | 114.1956 | 26.5508 |
| 22 | 109.0032 | 33.8302 | 96 | 103.2218 | 30.4787 | 170 | 115.9359 | 29.5364 |
| 23 | 109.0524 | 33.8561 | 97 | 104.0345 | 31.3827 | 171 | 114.9125 | 25.9242 |
| 24 | 110.3764 | 33.9539 | 98 | 103.8000 | 31.2424 | 172 | 117.3667 | 27.9167 |
| 25 | 107.7637 | 34.1653 | 99 | 107.2681 | 30.7237 | 173 | 114.0883 | 25.9617 |
| 26 | 107.8217 | 34.0617 | 100 | 106.9674 | 32.5970 | 174 | 116.9108 | 27.0684 |
| 27 | 107.7636 | 34.1701 | 101 | 107.4057 | 31.4865 | 175 | 114.2286 | 26.5448 |
| 28 | 110.3764 | 33.9539 | 102 | 102.2810 | 29.8787 | 176 | 115.9330 | 29.5207 |
| 29 | 107.7222 | 34.0958 | 103 | 103.5582 | 30.8566 | 177 | 116.0011 | 29.5615 |
| 30 | 108.6206 | 33.5741 | 104 | 101.8873 | 29.5994 | 178 | 115.2455 | 25.2546 |
| 31 | 106.8559 | 34.5801 | 105 | 103.6375 | 31.0961 | 179 | 115.1980 | 25.2737 |
| 32 | 106.8568 | 34.5812 | 106 | 103.4080 | 31.1682 | 180 | 115.1981 | 25.2755 |
| 33 | 115.8311 | 29.5036 | 107 | 119.8705 | 30.6135 | 181 | 106.9399 | 29.3698 |
| 34 | 114.8571 | 28.0927 | 108 | 119.4551 | 30.3408 | 182 | 108.6740 | 28.6037 |
| 35 | 117.4208 | 27.9503 | 109 | 119.2161 | 27.9393 | 183 | 108.3824 | 29.2366 |
| 36 | 110.2105 | 29.3272 | 110 | 119.4551 | 30.3408 | 184 | 107.8457 | 29.0480 |
| 37 | 114.0036 | 26.5332 | 111 | 120.1553 | 30.2377 | 185 | 108.8614 | 29.4816 |
| 38 | 110.1002 | 26.4137 | 112 | 119.4657 | 30.3311 | 186 | 107.1345 | 29.0554 |
| 39 | 110.6086 | 29.3685 | 113 | 119.5673 | 30.4130 | 187 | 107.3720 | 29.2295 |
| 40 | 109.8875 | 28.8878 | 114 | 119.4389 | 30.4184 | 188 | 108.2148 | 29.1425 |
| 41 | 109.6009 | 27.9219 | 115 | 118.6472 | 28.3093 | 189 | 108.8842 | 29.3617 |
| 42 | 110.2069 | 28.8192 | 116 | 118.6270 | 28.3313 | 190 | 105.8966 | 29.5932 |
| 43 | 110.8465 | 26.4580 | 117 | 119.4370 | 30.3532 | 191 | 108.2818 | 29.7425 |
| 44 | 109.4958 | 28.5983 | 118 | 119.4554 | 30.3408 | 192 | 105.8881 | 29.5375 |
| 45 | 110.1057 | 28.6521 | 119 | 108.5650 | 26.3769 | 193 | 109.4464 | 31.6591 |
| 46 | 109.9055 | 28.7604 | 120 | 104.3573 | 26.9686 | 194 | 107.5578 | 29.2575 |
| 47 | 110.0589 | 28.3945 | 121 | 107.3440 | 28.2657 | 195 | 107.2318 | 29.1402 |
| 48 | 109.2907 | 28.5070 | 122 | 107.2472 | 28.1966 | 196 | 107.1344 | 29.0552 |
| 49 | 110.1002 | 26.4137 | 123 | 109.2639 | 28.2387 | 197 | 108.6911 | 28.6271 |
| 50 | 110.7637 | 28.3368 | 124 | 109.1776 | 26.6728 | 198 | 111.1270 | 31.7223 |
| 51 | 109.5937 | 28.3451 | 125 | 108.1566 | 27.3408 | 199 | 109.1397 | 30.0366 |
| 52 | 110.2046 | 28.8054 | 126 | 105.3557 | 25.9913 | 200 | 110.4924 | 31.8175 |
| 53 | 110.7637 | 28.3368 | 127 | 106.2437 | 26.2074 | 201 | 110.3875 | 31.4427 |
| 54 | 110.4430 | 28.7653 | 128 | 106.3558 | 28.2961 | 202 | 110.6146 | 31.6670 |
| 55 | 112.7331 | 27.2733 | 129 | 107.7318 | 25.5756 | 203 | 110.9966 | 31.6526 |
| 56 | 114.0036 | 26.5332 | 130 | 103.8616 | 26.8662 | 204 | 110.0637 | 29.8878 |
| 57 | 109.4767 | 28.0472 | 131 | 107.6141 | 29.1579 | 205 | 112.1207 | 29.7770 |
| 58 | 110.8465 | 26.4580 | 132 | 108.3555 | 26.3017 | 206 | 109.9766 | 30.3517 |
| 59 | 110.2173 | 26.1894 | 133 | 107.2063 | 28.2462 | 207 | 110.3452 | 31.4343 |
| 60 | 119.4365 | 30.3538 | 134 | 108.1199 | 27.1091 | 208 | 115.7219 | 30.7607 |
| 61 | 119.5673 | 30.4130 | 135 | 107.6233 | 25.9245 | 209 | 114.5834 | 29.3755 |
| 62 | 119.4389 | 30.4184 | 136 | 108.3090 | 25.5998 | 210 | 113.8898 | 29.0717 |
| 63 | 119.4543 | 30.3190 | 137 | 106.3617 | 28.5992 | 211 | 114.6615 | 29.3970 |
| 64 | 119.4353 | 30.3411 | 138 | 108.7661 | 28.8483 | 212 | 110.4428 | 34.4955 |
| 65 | 112.1311 | 35.2543 | 139 | 104.1455 | 28.5251 | 213 | 106.5448 | 35.1904 |
| 66 | 112.1311 | 35.2543 | 140 | 102.6303 | 24.9763 | 214 | 105.2887 | 32.7139 |
| 67 | 111.6396 | 33.6482 | 141 | 99.0044 | 23.3256 | 215 | 104.5974 | 32.9056 |
| 68 | 110.4428 | 34.4955 | 142 | 103.0683 | 27.3230 | 216 | 111.9694 | 35.5929 |
| 69 | 110.4428 | 34.4955 | 143 | 118.1717 | 30.1437 | 217 | 111.8787 | 35.2961 |
| 70 | 115.0734 | 31.6203 | 144 | 118.0223 | 29.8331 | 218 | 111.9694 | 35.5929 |
| 71 | 112.1512 | 35.2683 | 145 | 116.2267 | 31.2326 | 219 | 117.6879 | 27.7537 |
| 72 | 110.8836 | 34.5392 | 146 | 118.9795 | 30.3955 | 220 | 118.2690 | 26.2473 |
| 73 | 110.8835 | 34.5389 | 147 | 115.7853 | 31.1411 | 221 | 115.9023 | 25.0038 |
| 74 | 100.9394 | 24.5363 | 148 | 115.7664 | 31.1641 | 222 | 111.0220 | 25.5394 |

**Table S2.** Information of 47 environment variables used in model prediction

| Abbreviation | Full name | Unit | Explanation |
| --- | --- | --- | --- |
| BIO01 | Annual Mean Temperature | °C/10 | Mean annual daily temperatures averaged over 1 year |
| BIO02 | Mean Diurnal Range (Mean of monthly (max temp - min temp)) | °C/10 | Mean diurnal range of temperatures averaged over 1 year |
| BIO03 | Isothermality (BIO2/BIO7) (×100) | °C/10 | Ratio of diurnal to annual variation in temperatures |
| BIO04 | Temperature Seasonality (standard deviation ×100) | °C/10 | Standard deviation of monthly mean temperatures |
| BIO05 | Max Temperature of Warmest Month | °C/10 | The highest value of any monthly daily maximum temperature |
| BIO06 | Min Temperature of Coldest Month | °C/10 | The lowest value of any monthly daily maximum temperature |
| BIO07 | Temperature Annual Range (BIO5-BIO6) | °C/10 | The difference between the maximum temperature of the warmest month and the minimum temperature of the coldest month |
| BIO08 | Mean Temperature of Wettest Quarter | °C/10 | The wettest quarter of the year is determined (to the nearest month) |
| BIO09 | Mean Temperature of Driest Quarter | °C/10 | The driest quarter of the year is determined (to the nearest month) |
| BIO10 | Mean Temperature of Warmest Quarter | °C/10 | The warmest quarter of the year is determined (to the nearest month) |
| BIO11 | Mean Temperature of Coldest Quarter | °C/10 | The coldest quarter of the year is determined (to the nearest month) |
| BIO12 | Annual precipitation | kg m^-2^ | Accumulated precipitation over 1 year |
| BIO13 | Precipitation of the wettest month | kg m^-2^ | The highest monthly precipitation |
| BIO14 | Precipitation of the driest month | kg m^-2^ | The lowest monthly precipitation |
| BIO15 | Precipitation seasonality (Coefficient of Variation) | kg m^-2^ | The coefficient of variation (i.e., standard deviation) of monthly precipitation estimates expressed as a percentage of the annual mean |
| BIO16 | Precipitation of Wettest Quarter | kg m^-2^ | The wettest quarter of the year is determined (to the nearest month) |
| BIO17 | Precipitation of Driest Quarter | kg m^-2^ | The driest quarter of the year is determined (to the nearest month) |
| BIO18 | Precipitation of Warmest Quarter | kg m^-2^ | The warmest quarter of the year is determined (to the nearest month) |
| BIO19 | Precipitation of Coldest Quarter | kg m^-2^ | The coldest quarter of the year is determined (to the nearest month) |
| NPP | Net primary productivity | g C m^-2^ y^-1^ 10^-1^ | Net primary productivity calculated based on the Miami model (Lieth, H., 1972) |
| SCD | Snow cover days | count | The number of days with snow cover calculated using the snowpack model implementation in TREELIM (https://doi.org/10.1007/s00035-014-0124-0) |
| annualPET | Annual potential evapotranspiration | mm year^-1^ | A measure of the ability of the atmosphere to remove water through evapotranspiration processes, given unlimited moisture |
| aridityIndexThornthwaite | Thornthwaite aridity index | — | Index of the degree of water deficit below water need |
| climaticMoistureIndex | Climatic moisture index | — | A metric of relative wetness and aridity |
| continentality | Continentality | °C | Mean temperature of the warmest month – mean temperature of the coldest month |
| embergerQ | Emberger's pluviothermic quotient | — | A metric designed to differentiate among Mediterranean climates |
| growingDegDays0 | Growing degree-days 0 | days | Sum of mean monthly temperature for months with mean temperature greater than 0℃ multiplied by the number of days |
| growingDegDays5 | Growing degree-days 5 | days | Sum of mean monthly temperature for months with mean temperature greater than 5℃ multiplied by the number of days |
| maxTempColdest | Maximum temperature of the coldest month | ℃ * 10 | Maximum temperature of the coldest month |
| meanTempColdest | Mean temperature of the coldest month | ℃ * 10 | Mean temperature of the coldest month |
| meanTempWarmest | Mean temperature of the warmest month | ℃ * 10 | Mean temperature of the warmest month |
| minTempWarmest | Minimum temperature of the warmest month | ℃ * 10 | Minimum temperature of the warmest month |
| PETColdestQuarter | Mean monthly potential evapo-transpiration of the coldest quarter | mm / month | Mean monthly potential evapo-transpiration of coldest quarter |
| PETDriestQuarter | Mean monthly potential evapo-transpiration of the driest quarter | mm / month | Mean monthly potential evapo-transpiration of driest quarter |
| PETseasonality | Monthly variability in potential evapotranspiration | mm / month | Monthly variability in potential evapotranspiration |
| PETWarmestQuarter | Mean monthly potential evapo-transpiration of the warmest quarter | mm / month | Mean monthly potential evapo-transpiration of the warmest quarter |
| PETWettestQuarter | Mean monthly potential evapo-transpiration of the wettest quarter | mm / month | Mean monthly potential evapo-transpiration of the wettest quarter |
| thermicityIndex | Compensated thermicity index | ℃ | (Sum of mean annual temperature, minimum temperature of the coldest month, and maximum temperature of the coldest month) * 10, with compensations for better comparability across the globe |
| aspectcosine | Aspect cosine | — | Topography |
| aspectsine | Aspect sine | — |  |
| elevation | Elevation | m |  |
| roughness | Roughness | — |  |
| slope | Slope | ° |  |
| TPI | Topographic position index | — |  |
| TRI | Terrain ruggedness index | — |  |
| VRM | Vector ruggedness measure | — |  |
| AvgSoilSedimDeposThick | Average soil and sedimentary deposit thickness | m | Average soil and sedimentary deposit thicknesses across upland hillslopes and valley bottoms in meters |

Lieth, H., 1972. Modelling the primary productivity of the earth. Nature and resources, UNESCO, VIII, 2:5-10.

**Table S3.** GC-MS data of 74 medicinal compounds identified in Schisandrae Sphenantherae Fructus

| No. | Retention time (t/min) | Compound name |
| --- | --- | --- |
|  |  |  |
| 1 | 4.59 | 2-Benzyloxyethylamine |
| 2 | 4.69 | Ethylbenzene |
| 3 | 4.82 | Phenelzine |
| 4 | 4.85 | Benzene, 1,3-dimethyl- |
| 5 | 4.98 | *p*-Xylene |
| 6 | 6.12 | Disiloxane,1,3-diethoxy-1,1,3,3-tetramethyl- |
| 7 | 6.73 | Tetraethyl silicate |
| 8 | 6.75 | trisiloxane,1,1,1,5,5,5-hexamethyl-3-[(trimethylsilyl)oxy]- |
| 9 | 7.32 | Diethyl sulfate |
| 10 | 7.64 | Methane, nitroso- |
| 11 | 9.04 | Silicic acid, diethyl bis(trimethylsilyl) ester |
| 12 | 9.43 | Silane, ethoxydimethylphenyl- |
| 13 | 11.16 | Silane, triethylmethoxy- |
| 14 | 11.32 | Silane, dodecyldiethoxymethyl- |
| 15 | 11.45 | Cyclopentasiloxane, decamethyl- |
| 16 | 14.40 | 1,1,3,3,5,5,7,7-Octamethyl-7-(2-methylpropoxy)tetrasiloxan-1-ol |
| 17 | 14.66 | Alpha-copaene |
| 18 | 14.67 | Ylangene |
| 19 | 15.86 | Cyclohexasiloxane, dodecamethyl- |
| 20 | 15.88 | Tricyclo[2.2.1.0(2,6)]heptane,1,7-dimethyl-7-(4-methyl-3-pentenyl)-, (-)- |
| 21 | 16.04 | Caryophyllene |
| 22 | 17.05 | Dimethyl phthalate |
| 23 | 17.65 | (1S,2E,6E,10R)-3,7,11,11-Tetramethylbicyclo[8.1.0]undeca-2,6-diene |
| 24 | 18.31 | Spiro[5.5]undec-2-ene,3,7,7-trimethyl-11-methylene-, (-)- |
| 25 | 18.42 | Alpha-copaene |
| 26 | 19.11 | Butylated hydroxytoluene |
| 27 | 19.22 | 1H-Benzocycloheptene,2,4a,5,6,7,8-hexahydro-3,5,5,9-tetramethyl-,(R)- |
| 28 | 19.44 | Benzene,1-methyl-4-(1,2,2-trimethylcyclopentyl)-,(R)- |
| 29 | 19.51 | (1S,4aR,8aS)-1-Isopropyl-7-methyl-4-methylene-1,2,3,4,4a,5,6,8a-octahydronaphthalene |
| 30 | 19.62 | Alpha -Muurolene |
| 31 | 19.81 | 1-Isopropyl-4,7-dimethyl-1,2,3,5,6,8a-hexahydronaphthalene |
| 32 | 19.83 | Naphthalene,1,2,3,5,6,8a-hexahydro-4,7-dimethyl-1-(1-methylethyl)-, (1S-cis)- |
| 33 | 20.40 | (1R,4S,5S)-1,8-Dimethyl-4-(prop-1-en-2-yl) spiro[4.5]dec-7-ene |
| 34 | 21.45 | 4-Hydroxybenzoic acid, 2TMS derivative |
| 35 | 29.02 | Cycloheptasiloxane, tetradecamethyl- |
| 36 | 29.19 | Pentasiloxane,1,1,3,3,5,5,7,7,9,9-decamethyl- |
| 37 | 29.20 | N-Ethyl-2-phenyl-N-(pyridin-4-ylmethyl)-3-[(trimethylsilyl)oxy]propanamide |
| 38 | 32.05 | Octadecane, 3-ethyl-5-(2-ethylbutyl)- |
| 39 | 32.43 | Ylangenal |
| 40 | 33.06 | (1S,4R,5S)-1-Methyl-4-(prop-1-en-2-yl)spiro[4.5]dec-7-ene-8-carbaldehyde |
| 41 | 33.61 | (1aR,4aS,8aS)-4a,8,8-Trimethyl-1,1a,4,4a,5,6,7,8-octahydrocyclopropa[d]naphthalene-2-carbaldehyde |
| 42 | 34.77 | Hexasiloxane,1,1,3,3,5,5,7,7,9,9,11,11-dodecamethyl- |
| 43 | 35.33 | 1,1,3,3,5,5,7,7,9,9-Decamethyl-9-(2-methylpropoxy)pentasiloxan-1-ol |
| 44 | 35.61 | *n-*Hexadecanoic acid |
| 45 | 35.65 | Cyclooctasiloxane, hexadecamethyl- |
| 46 | 36.60 | (5á)Pregnane-3,20á-diol,14à,18à-[4-methyl-3-oxo-(1-oxa-4-azabutane-1,4-diyl)]-, diacetate |
| 47 | 36.64 | Dibutyl phthalate |
| 48 | 37.17 | Hexadecanoic acid, ethyl ester |
| 49 | 38.02 | Heptasiloxane,1,1,3,3,5,5,7,7,9,9,11,11,13,13-tetradecamethyl- |
| 50 | 38.05 | Cyclononasiloxane, octadecamethyl- |
| 51 | 38.78 | 2,8,9-Trioxa-5-aza-1-silabicyclo(3.3.3)undecane, 1-methoxy- |
| 52 | 39.07 | 9,12-Octadecadienoic acid, ethyl ester |
| 53 | 39.14 | 7,15-Dihydroxydehydroabietic acid, methylester,di(trimethylsilyl)ether |
| 54 | 39.20 | Octasiloxane,1,1,3,3,5,5,7,7,9,9,11,11,13,13,15,15-hexadecamethyl- |
| 55 | 39.50 | Heptadecanoic acid, 15-methyl-, ethyl ester |
| 56 | 40.37 | Cyclodecasiloxane, eicosamethyl- |
| 57 | 40.61 | Corticosteron, 2TMS derivative |
| 58 | 40.76 | 1-Cyclohexyldimethylsilyloxy-3,5-dimethylbenzene |
| 59 | 41.20 | 9-Octadecenamide, (Z)- |
| 60 | 41.53 | 9-Desoxo-9-x-acetoxy-3-desoxy-7.8.12-tri-O-acetylingol-3-one |
| 61 | 41.77 | Phenol,2,2'-methylenebis[6-(1,1-dimethylethyl)-4-methyl- |
| 62 | 42.52 | 3-Hydroxypropyl palmitate, TMS derivative |
| 63 | 42.64 | Methyl glycocholate, 3TMS derivative |
| 64 | 42.80 | 4H-Cyclopropa[5',6']benz[1',2':7,8]azuleno[5,6-b]oxiren-4-one,8,8a-bis(acetyloxy)-2a-[(acetyloxy)methyl]-1,1a,1b,1c,2a,3,3a,6a,6b,7,8,8a-dodecahydro-6b-hydroxy-3a-methoxy-1,1,5,7-tetramethyl-,[1aR-(1aà,1bá,1cà,2aà,3aá,6aà,6bà,7à,8á,8aà)]- |
| 65 | 43.04 | 9-Desoxo-9x-hydroxy-7-ketoingol3,8,9,12-tetraacetate |
| 66 | 43.36 | Phthalic acid, di(2-propylpentyl) ester |
| 67 | 43.39 | Diisooctyl phthalate |
| 68 | 44.45 | (22R)-6à,11á,21-Trihydroxy-16à,17à-propylmethylenedioxypregna-1,4-diene-3,20-dione |
| 69 | 45.41 | 5,5'-((2R,3S)-2,3-Dimethylbutane-1,4-diyl)bis(benzo[d][1,3]dioxole) |
| 70 | 45.51 | Colchicine,7-acetoxy-7-desacetamino-1O-methio-10-desmethoxy- |
| 71 | 45.54 | 19-Norethandrolone, TBDMS derivative |
| 72 | 45.96 | 4-((2S,3R)-4-(Benzo[d][1,3]dioxol-5-yl)-2,3-dimethylbutyl)-2-methoxyphenol |
| 73 | 47.39 | 3-Nor-3,7-secodichotine,2,7-didehydro-2-deoxy-14-hydroxy-25-oxo-,(14à)- |
| 74 | 49.08 | 1,1',7a,7a'-Tetrahydro-6,6'-dimethoxy-1a,1a'-bi-(1aH)cyclopropa[b]naphthalene-2,2',7,7'-tetrone |

**Table S4.** Site information and secondary metabolites content of 32 sampling sites in Schisandrae Sphenantherae Fructus

|  | Longitude/E | Latitude/N | Volatile oils | Polysaccharides | Schisandrol A | Gomisin J | Schisantherin B | Schisanhenol | Schisandrin A | Schisandrin B | Schisandrin C | Schisandrol B | Schisantherin A |
| --- | --- | --- | --- | --- | --- | --- | --- | --- | --- | --- | --- | --- | --- |
| S1 | 108.5879 | 33.7896 | 0.133 | 1.850 | 6.781 | 0.861 | 1.231 | 0.512 | 5.111 | 2.962 | 0.242 | 0.273 | 0.975 |
| S2 | 109.1385 | 33.4391 | 0.124 | 3.070 | 0.000 | 0.000 | 0.728 | 0.382 | 5.496 | 0.000 | 0.304 | 0.097 | 1.355 |
| S3 | 108.5645 | 34.1243 | 0.144 | 9.280 | 0.000 | 0.000 | 1.092 | 0.688 | 8.702 | 0.025 | 0.232 | 0.090 | 3.956 |
| S4 | 110.2707 | 34.4922 | 0.133 | 5.760 | 0.030 | 0.005 | 1.531 | 0.520 | 4.803 | 0.093 | 0.091 | 0.038 | 3.074 |
| S5 | 105.7554 | 34.5706 | 0.155 | 9.810 | 0.472 | 0.043 | 0.109 | 0.136 | 0.461 | 0.230 | 0.019 | 0.000 | 0.097 |
| S6 | 112.1409 | 32.0103 | 0.149 | 7.510 | 0.039 | 0.002 | 0.080 | 0.058 | 0.384 | 0.372 | 0.010 | 0.030 | 0.122 |
| S7 | 106.8614 | 32.3718 | 0.153 | 8.660 | 0.049 | 0.013 | 0.036 | 0.340 | 0.270 | 0.022 | 0.043 | 0.000 | 0.036 |
| S8 | 107.5024 | 33.2039 | 0.139 | 6.180 | 0.010 | 0.011 | 0.172 | 3.144 | 1.287 | 0.029 | 0.720 | 0.000 | 0.304 |
| S9 | 108.1947 | 33.208 | 0.142 | 13.110 | 0.001 | 0.011 | 0.015 | 0.023 | 0.012 | 0.517 | 0.014 | 0.010 | 0.055 |
| S10 | 104.8342 | 31.7529 | 0.164 | 6.390 | 0.033 | 0.006 | 0.512 | 7.369 | 3.397 | 0.123 | 1.791 | 0.042 | 0.044 |
| S11 | 104.5084 | 32.2522 | 0.164 | 3.860 | 0.070 | 0.027 | 0.638 | 17.905 | 7.149 | 0.101 | 1.644 | 0.020 | 0.221 |
| S12 | 108.5088 | 32.8931 | 0.141 | 9.860 | 0.000 | 0.007 | 0.011 | 0.032 | 0.013 | 0.125 | 0.005 | 0.009 | 0.061 |
| S13 | 114.2186 | 30.5543 | 0.152 | 10.090 | 0.033 | 0.004 | 0.018 | 2.631 | 0.031 | 0.171 | 0.205 | 0.019 | 0.113 |
| S14 | 109.1292 | 33.6663 | 0.163 | 10.720 | 0.017 | 0.007 | 0.019 | 0.934 | 1.991 | 0.306 | 0.170 | 0.074 | 0.115 |
| S15 | 111.4736 | 33.3073 | 0.161 | 7.070 | 0.979 | 0.219 | 0.000 | 0.036 | 0.000 | 0.046 | 0.027 | 0.128 | 1.721 |
| S16 | 107.4578 | 34.0482 | 0.152 | 10.150 | 0.006 | 0.005 | 0.075 | 0.104 | 0.639 | 0.014 | 0.005 | 0.010 | 0.187 |
| S17 | 111.2831 | 31.895 | 0.162 | 7.700 | 0.042 | 0.003 | 0.096 | 0.025 | 0.460 | 0.239 | 0.011 | 0.033 | 0.150 |
| S18 | 111.033 | 34.008 | 0.154 | 8.710 | 0.243 | 0.059 | 1.387 | 0.543 | 6.240 | 0.181 | 0.039 | 0.071 | 2.776 |
| S19 | 108.3313 | 33.3628 | 0.167 | 5.390 | 0.053 | 0.027 | 1.203 | 0.401 | 6.117 | 0.146 | 0.248 | 0.115 | 2.184 |
| S20 | 106.0669 | 33.234 | 0.155 | 9.940 | 0.023 | 0.002 | 0.018 | 1.142 | 0.158 | 0.066 | 0.012 | 0.006 | 0.138 |
| S21 | 99.7136 | 27.8592 | 0.149 | 5.590 | 0.118 | 0.000 | 0.029 | 11.278 | 1.614 | 0.764 | 0.115 | 0.070 | 0.322 |
| S22 | 109.1829 | 31.7847 | 0.167 | 6.620 | 0.037 | 0.007 | 0.113 | 0.318 | 0.024 | 0.319 | 0.008 | 0.003 | 0.266 |
| S23 | 109.1355 | 33.6501 | 0.159 | 16.170 | 0.089 | 0.012 | 0.380 | 0.224 | 3.516 | 0.070 | 0.049 | 0.023 | 1.634 |
| S24 | 106.9495 | 33.5391 | 0.171 | 6.610 | 0.208 | 0.022 | 0.017 | 0.000 | 0.019 | 0.108 | 0.046 | 0.021 | 0.172 |
| S25 | 110.2397 | 34.4883 | 0.165 | 7.310 | 1.107 | 0.130 | 0.003 | 0.100 | 3.640 | 0.506 | 0.368 | 0.073 | 0.661 |
| S26 | 107.9724 | 33.3985 | 0.177 | 3.740 | 0.006 | 0.014 | 0.009 | 0.162 | 1.402 | 0.211 | 0.066 | 0.010 | 0.118 |
| S27 | 109.8823 | 33.5322 | 0.173 | 4.810 | 0.051 | 0.000 | 0.000 | 0.072 | 6.110 | 0.042 | 0.168 | 0.090 | 1.067 |
| S28 | 109.2144 | 33.4295 | 0.133 | 8.120 | 0.066 | 0.034 | 0.000 | 0.073 | 5.499 | 0.011 | 0.165 | 0.042 | 0.410 |
| S29 | 108.9316 | 32.1831 | 0.153 | 8.770 | 0.055 | 0.006 | 0.002 | 0.216 | 0.019 | 0.367 | 0.005 | 0.025 | 0.077 |
| S30 | 108.0722 | 33.2874 | 0.140 | 5.500 | 0.019 | 0.087 | 0.037 | 0.022 | 0.027 | 0.767 | 0.022 | 0.006 | 0.050 |
| S31 | 107.3871 | 33.2157 | 0.213 | 10.610 | 0.031 | 0.015 | 0.008 | 0.383 | 0.245 | 0.150 | 0.075 | 0.046 | 0.061 |
| S32 | 109.0867 | 33.8344 | 0.183 | 5.540 | 0.089 | 0.022 | 0.000 | 0.047 | 5.917 | 0.017 | 0.089 | 0.043 | 0.443 |
